# Association of childhood mild traumatic brain injury with mental and behavioral health outcomes later in life among U.S. Veterans: A LIMBIC-CENC study

**DOI:** 10.1371/journal.pmen.0000679

**Published:** 2026-10-21

**Authors:** Dana Waltzman, Juliet Haarbauer-Krupa, Samuel R. Walton, Amani Jridi, Angela P. Presson, William C. Walker, David X. Cifu, Jessie R. Oldham, Landon B. Lempke

**Affiliations:** 1 Department of Physical Medicine and Rehabilitation, Indiana University School of Medicine, Indianapolis, Indiana, United States of America; 2 Department of Pediatrics, Emory University School of Medicine, Atlanta, Georgia, United States of America; 3 Department of Physical Medicine and Rehabilitation, Virginia Commonwealth University School of Medicine, Richmond, Virginia, United States of America; 4 Richmond Veterans Affairs Medical Center, Central Virginia Veterans Affairs Health Care System, Richmond, Virginia, United States of America; 5 Institute for Women’s Health, Virginia Commonwealth University, Richmond, Virginia, United States of America; 6 Division of Epidemiology, The University of Utah, Salt Lake City, Utah, United States of America; 7 United States Department of Veterans Affairs, Brain Health Coordinating Center, Richmond, Virginia, United States of America; University of Padova: Universita degli Studi di Padova, ITALY

## Abstract

Mild traumatic brain injury (mTBI) is a common injury across all age groups and populations, with a high prevalence among military service members and Veterans. The purpose of this cross-sectional study was to use a large, prospective cohort of Veterans to examine the association between childhood mTBI and multimodal mental and behavioral health outcomes, and among those with childhood mTBI, evaluate sociodemographic characteristics associated with worse outcomes. Data came from the Long-term Impact of Military-relevant Brain Injury Consortium-Chronic Effects of Neurotrauma Consortium (LIMBIC-CENC) prospective longitudinal study. This cohort included 1,286 Veterans, 61.3% of whom sustained a childhood mTBI. After adjustment for demographic, behavioral, and military history factors, Veterans with childhood mTBI had higher PTSD symptom scores (Mean Ratio[MR] = 1.36, 95%CI: 1.22–1.51), higher anger scores (MR = 1.08, 95%CI: 1.05–1.10), higher depression scores (MR = 1.31, 95% CI: 1.17–1.46), higher anxiety scores (mean difference [MD]= 4.3, 95% CI: 2.9-5.6), emotional-behavioral dyscontrol scores (MD = 3.2, 95% CI: 2.2–4.3), worse sleep quality (MD = 1.6, 95% CI: 1.1-2.2), worse neurobehavioral symptoms (MD = 6.8, 95% CI: 5.2-8.5), lower satisfaction with life (MD = -1.2, 95% CI: -2.1 to -0.27), and lower resilience scores (MR = 0.96, 95% CI: 0.94–0.99). There were no significant differences after adjustment for alcohol or drug use scores. Among individuals with a history of childhood mTBI, several mental and behavioral health outcomes were worse among those who sustained multiple mTBIs and better among those who were physically active and had higher education. These findings suggest markers of increased riskpresent before military enlistment that may warrant targeted prevention, screening, and treatment strategies. Increased awareness among healthcare providers may help identify Veterans with childhood mTBI history who would benefit from evidence-based treatment and support.

## Introduction

Mild traumatic brain injury (mTBI) is a common injury across all age groups and populations, with leading causes among children being unintentional falls and sports- and recreation-related activities. mTBI is a significant health concern because of the immediate physical, cognitive, and mental burden and also due to the intermediate- and long-term health implications [1–4]. While clinical recovery (i.e., symptom resolution and normalization of function) from mTBI typically occurs within 4 weeks [5–7], a subset of patients experience chronic symptoms that persist for months or years after injury and can significantly impact their independence and quality of life [8–10]. One gap in the literature is limited research on the long-term consequences of sustaining a mTBI during childhood. From these few studies, there is evidence to suggest an association between sustaining a mTBI in childhood and adverse long-term outcomes later in life such as lower academic achievement, cardiovascular, endocrine, neurologic, and psychiatric disorders, disability pension, and welfare recipiency [11–15]. Additionally, prior research has shown a potential association with earlier repetitive head impact exposures and worse cognitive and behavioral outcomes along with greater mental health disorder risk [16–18]. These findings indicate the need for long term monitoring and potential need for better treatments to lessen chronicity and manage long term effects [9,10,19].

In the adult population, there is an especially high prevalence of mTBI among military service members and Veterans [20,21] who sustained more than 436,000 first medically documented military-related mTBIs from the years 2000–2025, reflecting only the initial documented injury per individual and may underestimate the cumulative health burden. Complicating factors for their mTBI recovery and outcomes include greater risk for many other adverse health conditions [22–25]. For example, Agimi and colleagues [22] found that among 42,000 service members with a first lifetime military mTBI, 77.6% had at least one comorbidity post-mTBI diagnosis, 42.9% had headache and related conditions (e.g., migraine or other primary/secondary headache syndromes), 36.3% had a sleep-related condition, 26.0% had posttraumatic stress disorder (PTSD), and 24.6% had cognitive conditions (e.g., memory impairment, disorientation, impulsivity, or speech/language disturbance). Overall, potential factors associated with poor mTBI outcomes among service members and Veterans are complex and include characteristics of the injury itself, age, pre-injury status, comorbid conditions, environment, and resilience traits [9].

Most research examining mTBI in military populations has focused on injuries sustained during military service, particularly those occurring during combat, training, or other service-related activities [22–26]. Consequently, substantially less is known about the potential long-term implications of mTBIs sustained before military service and whether these earlier injuries contribute independently to later mental and behavioral health outcomes. Childhood mTBI may represent a pre-military exposure that predates military training, deployment, combat exposure, and military-related injuries, potentially influencing health trajectories before service begins. Understanding the association between childhood mTBI and later-life outcomes among Veterans may therefore help clarify the role of pre-military factors in shaping long-term health and resilience. Despite the growing literature on military-related mTBI, it remains unclear how mTBI experienced prior to military service, particularly during childhood, contributes independently or cumulatively to these adverse health outcomes among U.S. service members and Veterans. This knowledge gap limits the development of effective prevention, monitoring, and treatment strategies for this population.

The purpose of this exploratory study was to assess the prevalence of childhood mTBI in former military service members (i.e., Veterans) and the relationship between childhood mTBI history and adverse health outcomes. Using a large prospective cohort of Veterans, this study analyzes the association between childhood mild TBI presence and multimodal mental and behavioral health outcomes, and among those with childhood mTBI, evaluate sociodemographic characteristics with worse mental and behavioral health outcomes.

## Methods

### Ethics statement

All participants provided written informed consent prior to participation, all LIMBIC-CENC sites had institutional review board approval by their respective IRB, and all study procedures were conducted in accordance with the Declaration of Helsinki.

Data came from the Long-term Impact of Military-relevant Brain Injury Consortium-Chronic Effects of Neurotrauma Consortium (LIMBIC-CENC) prospective longitudinal study (PLS) [27,28]. Briefly, the LIMBIC-CENC PLS is a multicenter clinical research study with the overarching aim of investigating the long-term effects of mTBI, blast, and other military exposures with multimodal assessments of brain, psychologic, and general health. Data from US service members and Veterans were collected from 10 LIMBIC-CENC PLS sites at 9 Veteran Affairs (VA) Medical Centers and 1 Military Treatment Facility, primarily through mass mailings, advertisements, and clinic referrals. Participant enrollment occurred between 2016 and 2025 for this report. Greater details about the study methodology are reported elsewhere [27,28].

Overarching LIMBIC-CENC PLS study eligibility criteria for participants were 18 years and older, current or former military, previous combat deployment with any combat exposure, and completed comprehensive assessments at their initial baseline visit. The only exclusion criterion was a history of moderate-to-severe TBI or a major neurologic or psychiatric disorder (e.g., stroke, spinal cord injury, bipolar disorder or schizophrenia). Specific to this report, we further excluded participants in the following stepwise manner from the initial n = 1,613 with an mTBI during childhood: n = 231 for invalid symptom reporting based on ≥ 8 on the Mild Brain Injury Atypical Symptom (mBIAS) scale [29], n = 8 for invalid neurocognitive performance based on < 7 on the Digit Span test from Wechsler Adult Intelligence Scale (WAIS-IV), n = 88 ≤ 93% on the Forced Choice from California Verbal Learning Test (CVLT-II) [30] resulting in a maximum sample size of n = 1,286. Further analysis-specific exclusion occurred based on variable missingness for each mental and behavioral health model independently.

### Measures

*Lifetime history of childhood mTBI and mTBI circumstances.* Lifetime mTBI was based on the VA/Department of Defense (VA/DoD) common definition of mTBI [31], which also aligns with the American Congress of Rehabilitation Medicine mTBI definition [32]. Lifetime mTBI was assessed through a multi-step process. First, a modified version of the Ohio State University (OSU) TBI Identification Method survey [33] was used to identify self-reported mTBI(s) and other potential concussive events. Next, a validated structured interviewing tool, the Virginia Commonwealth University retrospective Concussion Diagnostic Interview (VCU rCDI) [34], was used to generate a preliminary mTBI diagnosis for each of the identified potential concussive events. Each preliminary mTBI diagnosis was then reviewed by the site principal investigator, checked against any additional information and available medical records, and further reviewed by a centralized quality assurance process that included a consensus of an expert committee to determine a final clinical diagnosis [34,35].

For this report, lifetime mTBI history was parameterized to focus on mTBIs occurring during childhood (ages 1–17). This was dichotomized to create a binary variable of lifetime history of mTBI in childhood vs no lifetime childhood mTBI history. Since it was not the focus of the study, the initial sample (n = 2,541) that included those who sustained a mTBI only in adulthood was excluded (n = 1,255). Additionally, other mTBI circumstances were reported and used as covariates where appropriate and included cumulative lifetime mTBI history (i.e., combined adulthood and childhood; categorical), time since last mTBI (continuous, years), and age at the time of injury of first mTBI (continuous).

*Mental health outcomes*. A total of five assessments under the mental health domain were evaluated in this present report:

1. The PTSD Checklist for the Diagnostic and Statistical Manual of Mental Disorders, Fifth Edition (PCL-5) [36] was used where participants completed a 17-item inventory rated on a 5-point Likert scale to self-report the presence of PTSD-related symptoms over the past month. The total score was calculated by summing all 17 items (range 0–85) with a higher score indicating higher PTSD-related symptom presence.
2. The Traumatic Brain Injury Quality of Life (TBI-QoL) [37] Anger domain included 10 items scored on a 5-point Likert scale where Veterans self-reported anger symptoms within the past 7 days. Total scores were calculated by summing all 10 items (range 10–50) and then transformed into T-scores (mean = 50, standard deviation = 10) via item response theory and established guidelines [37,38], with higher scores indicating greater anger.
3. The TBI-QoL [37] Anxiety domain consisted of 10 items scored on a 5-point Likert scale to self-report anxiety symptoms within the past 7 days. Scoring followed identical procedures to the TBI-QoL Anger domain, with higher T-scores indicating greater anxiety.
4. The Patient Health Questionnaire-9 (PHQ-9) [39] consisted of 9 items where Veterans rated their depressive symptomology over the past 2 weeks on a 4-point Likert scale. The total score was calculated as the sum of all 9 items (range 0–27) with higher scores reflecting higher depressive symptoms.
5. The Satisfaction with Life Scale (SWLS) [40] is a 5-item questionnaire using a 7-point Likert scale where Veterans reported their level of agreement with life satisfaction statements reflecting on their life as a whole. The total score was calculated by summing across all 5 items (range 5–35) with higher scores indicating greater trait-based life satisfaction.

*Behavioral health outcomes*. Six reliable and valid assessments under the behavioral health domain were evaluated for this report:

1. The TBI-QoL [37] emotional-behavioral dyscontrol domain consisted of 10 items on a 5-point Likert scale to self-report emotional-behavioral dyscontrol symptoms within the past 7 days. Scoring followed identical procedures to the TBI-QoL Anger domain, with higher scores indicating greater emotional-behavioral dyscontrol.
2. The TBI-QoL [37] Resilience domain consisted of 10 items on a 5-point Likert scale to self-report resilience symptoms within the past 7 days. Scoring followed identical procedures to the TBI-QoL Anger domain with higher scores indicating worse resiliency.
3. The Alcohol Use Disorder Identification Test - Consumption (AUDIT-C) [41] was a 3-item questionnaire on a 5-point Likert scale to self-report alcohol use within the past year. Total scores were calculated by summing all 3 items (range 0–12) with higher scores indicating greater alcohol use.
4. The Drug Abuse Screening Test-10-item (DAST-10) [42] questionnaire asked Veterans to dichotomously (yes/no) report illicit substances and non-medical drug use across specified drug classes. The total score was calculated by summing number of items where the response was affirmative (range 0–10) with higher scores reflecting greater illicit drug use.
5. The Pittsburgh Sleep Quality Index (PSQI) [43] was a 19-item questionnaire consisting of a of Likert scale and open-ended questions to assess subjective sleep quality over the past month. The total score was calculated from the 7 component scores the PSQI captures (subjective sleep quality, sleep latency, sleep duration, habitual sleep efficiency, sleep disturbances, sleep medication use, daytime dysfunction), and represents the sum of all 19 items (range 1–21) with higher scores indicating poorer quality of sleep.
6. The 22-item Neurobehavioral Symptom Inventory (NSI) [44] had Veterans report common post-mTBI symptoms on a 4-point Likert scale based on how much they have disturbed them in the past 2 weeks. The total score was derived from summing the subscale scores across 4 symptom domains (somatic, cognitive, affective, and vestibular; range 0–88) with higher scores reflecting greater mTBI-related symptoms.

Additional demographic and military characteristics were also collected from participants. Specifically, participants reported their age, body mass index (BMI; participants reported height and weight from which BMI was calculated), sex, race, housing type, marital status, and number of years they served in the military. Additionally, participants completed the Centers for Disease Control and Prevention’s Behavioral Risk Factor Surveillance System questionnaire [45] to evaluate their smoking and chewing dip use, and physical activity. Smoking and chewing dip use were both binarily classified where participants we classified as “yes” if they reported use everyday or some days, and as “no” if they reported not at all. Physical activity specifically was binarily classified based on whether participants did or did not meet moderate to vigorous aerobic exercise guidelines set by the Department of Health and Human Services (DHHS) as previously described [46–48].

### Statistical analyses

Descriptive statistics were calculated to describe demographic characteristics and mental and behavioral health outcomes for the sample and stratified by childhood mTBI group. Bivariate tests were used to descriptively report differences between lifetime childhood mTBI group and demographics and mental and behavioral outcomes. Continuous variables with approximately symmetric distributions were compared using the Welch two-sample t-test. Variables with markedly skewed distributions were compared using the Wilcoxon rank-sum test. Categorical variables were compared using the Pearson chi-square test or Fisher’s exact test when any expected cell count was < 5.

In this cross-sectional analysis, separate multivariable linear, gamma, quasi-Poisson, or ordinal logistic regressions were used to estimate the association between lifetime childhood mTBI group and each mental and behavioral health outcome, with model selection based on the distributional characteristics of the outcome.. Most outcomes (anxiety, emotional behavioral dyscontrol score, neuro-behavioral symptoms score, satisfaction with life score, sleep quality score) were analyzed using linear regression, with results reported as mean differences and 95% confidence intervals (CI). For non-normally distributed outcomes, the modified Park test was used to help identify an appropriate variance function and guide selection among candidate generalized linear models [49]. Gamma regression with a log link was used to model anger and resilience, which were positively skewed with variances increasing with the mean, and the results are reported as mean ratios (MR). A log link was used so that results are reported as mean ratios (MR). Quasi-Poisson regression with a log link was used for PTSD symptoms, depression severity, and alcohol use score because these outcomes exhibited overdispersion (variance exceeding the mean);results were also reported as MR. Drug use severity was analyzed using ordinal logistic regression, with results were reported as odds ratios (OR). The proportional odds assumption was assessed for the multivariable model for drug use severity and was found to be satisfied. In each multivariable model, the main exposure variable was childhood mTBI, with the no lifetime childhood mTBI group as the reference, and adjustment variables included age (continuous), body mass index (BMI; continuous), education (categorical), sex (binary), race (categorical), smoking status (binary), chew dip status (binary), physical activity (binary), housing type (dichotomous), marital status (dichotomous), and years in military (continuous). Missing data were handled using complete-case (case-wise deletion) analysis. Univariate models were also conducted for completeness and presented in supplemental material (S1 Table). All statistical analyses were performed in R software, version 4.5.1(R Foundation for Statistical Computing, Vienna, Austria). All tests were two-tailed and statistical significance was evaluated at p < 0.05.

## Results

### Study sample characteristics

Detailed demographic characteristics of the LIMBIC-CENC PLS Veteran sample included in this study can be found in Table 1. Briefly, this cross-sectional cohort included 1,286 adults who were predominantly male (85.5%) and with a mean age of 43 years. A little over half (52.2%) of the Veterans had a Bachelor’s degree or higher. A majority of the sample were White (76.5%), married (59.4%), had 13.1 ± 9.0 years of military experience, and had sustained a mTBI (61.3%) during childhood in their lifetime. Among Veterans who sustained a mTBI during childhood, 83.2% had multiple mTBIs (2 or more) and was first sustained when they at 11.4 ± 4.0 mean years of age. A summary of demographics and mental and behavioral health outcomes for the sample and by lifetime childhood mTBI status are also provided in Table 1.

**Table 1:** Characteristics of the Long-term Impact of Military-relevant Brain Injury Consortium-Chronic Effects of Neurotrauma Consortium (LIMBIC-CENC) study sample.

Characteristic	Lifetime history of childhood mTBI (N = 788)	No lifetime mTBI history(N = 498)	Total(N = 1286)
**Sex**			
Female	76 (9.7%)	110 (22.1%)	186 (14.5%)
Male	711 (90.3%)	388 (77.9%)	1099 (85.5%)
Missing	1 (0.13%)	–	1 (0.078%)
**Age (years)**			
Mean(SD)	42.4 (10.4)	43.8 (11.6)	43.0 (10.9)
Median (IQR)	41.0 (34.0, 50.0)	43.0 (35.0, 51.0)	41.0 (35.0, 50.0)
**Education**			
High School or Less	80 (10.2%)	57 (11.4%)	137 (10.7%)
Some College	307 (39.0%)	171 (34.3%)	478 (37.2%)
Bachelor’s Degree or more	401 (50.9%)	270 (54.2%)	671 (52.2%)
**Race**			
White	633 (81.6%)	338 (68.4%)	971 (76.5%)
Black	88 (11.3%)	112 (22.7%)	200 (15.7%)
Other	32 (4.1%)	19 (3.8%)	51 (4.0%)
Asian	9 (1.2%)	14 (2.8%)	23 (1.8%)
American Indian or Alaska Native	11 (1.4%)	4 (0.8%)	15 (1.2%)
Pacific Islander	3 (0.4%)	7 (1.4%)	10 (0.8%)
Missing	12 (1.52%)	4 (0.8%)	16 (1.2%)
**Marital Status**			
Married	479 (61.1%)	282 (56.6%)	761 (59.4%)
Divorced	139 (17.7%)	106 (21.3%)	245 (19.1%)
Widowed	7 (0.9%)	3 (0.6%)	10 (0.8%)
Separated	23 (2.9%)	18 (3.6%)	41 (3.2%)
Never Married	118 (15.1%)	77 (15.5%)	195 (15.2%)
Unmarried Couple	18 (2.3%)	12 (2.4%)	30 (2.3%)
Missing	4 (0.50%)	–	4 (0.31%)
**BMI**			
Mean(SD)	30.1 (5.2)	29.7 (5.3)	29.9 (5.3)
Median (IQR)	29.3 (26.6, 33.1)	29.2 (26.0, 32.6)	29.3 (26.4, 32.9)
Missing	8 (1.0%)	5 (1.0%)	13 (1.0%)
**Smoking Status**			
Yes	127 (16.1%)	70 (14.1%)	197 (15.3%)
No	661 (83.9%)	428 (85.9%)	1089 (84.7%)
**Chew Dip Status**			
Yes	77 (9.8%)	14 (2.8%)	91 (7.1%)
No	710 (90.2%)	480 (97.2%)	1190 (92.9%)
Missing	1 (0.13%)	4 (0.8%)	5 (0.39%)
**Physical Activity**			
Yes	655 (83.3%)	409 (82.8%)	1064 (83.1%)
No	131 (16.7%)	85 (17.2%)	216 (16.9%)
Missing	1 (0.13%)	4 (0.8%)	6 (0.47%)
**Housing Type**			
Private residence	776 (99.2%)	495 (99.4%)	1271 (99.3%)
Group home	5 (0.6%)	2 (0.4%)	7 (0.5%)
Homeless shelter	1 (0.1%)	1 (0.2%)	2 (0.2%)
Missing	5 (0.63%)	–	5 (0.39%)
**Years in the Military**			
Mean(SD)	12.9 (9.0)	13.5 (9.1)	13.1 (9.0)
Median (IQR)	9.0 (5.0, 20.0)	10.0 (5.0, 22.0)	9.0 (5.0, 21.0)
Missing	173 (22.0%)	69 (14%)	242 (19%)
**Lifetime childhood mTBI**			
Yes	–	–	788 (61.3%)
No	–	–	498 (38.7%)
Cumulative Lifetime mTBI*			
1	132 (16.8%)	–	–
2	202 (25.6%)	–	–
3+	454 (57.6%)	–	–
Cumulative Childhood mTBI*			
1	565 (71.7%)	–	–
2	159 (20.2%)	–	–
3+	64 (8.1%)	–	–
Time Since Last TBI (Years)*			
Mean(SD)	16.0 (12.4)	–	–
Median (IQR)	12.7 (7.3, 21.1)	–	–
Age of First mTBI (Years)*			
Mean(SD)	11.4 (4.0)	–	–
Median (IQR)	12.0 (8.0, 15.0)	–	–
**mTBI Mechanism**			
Blast	2 (0.3%)		
Not Blast	786 (99.7%)		
**PTSD Symptoms (PCL-5)**			
Mean(SD)	23.1 (17.1)	18.0 (16.8)	21.1 (17.2)
Median (IQR)	21.0 (9.0, 34.8)	12.0 (4.0, 27.0)	17.0 (7.0, 32.0)
Missing	2 (0.25%)	–	2 (0.16%)
**Anger (TBI-QoL)**			
Mean(SD)	53.1 (9.8)	49.7 (9.9)	51.8 (10.0)
Median (IQR)	53.3 (45.2, 59.5)	47.4 (42.5, 56.6)	50.7 (45.2, 58.5)
Missing	71 (9.0%)	52 (10.0%)	123 (9.6%)
**Anxiety (TBI-QoL)**			
Mean(SD)	53.7 (10.1)	49.8 (10.4)	52.2 (10.4)
Median (IQR)	55.3 (46.1, 61.4)	49.4 (41.2, 57.1)	52.0 (43.9, 59.7)
Missing	69 (8.8%)	54 (11.0%)	123 (9.6%)
**Depression (PHQ-9)**			
Mean(SD)	7.3 (5.7)	5.7 (5.5)	6.7 (5.6)
Median (IQR)	6.0 (3.0, 11.0)	4.0 (1.0, 9.0)	5.0 (2.0, 10.0)
Missing	4 (0.51%)	4 (0.8%)	8 (0.62%)
**Satisfaction with Life (SWLS)**			
Mean(SD)	21.9 (7.7)	22.7 (7.4)	22.2 (7.6)
Median (IQR)	23.0 (16.0, 28.0)	24.0 (18.0, 29.0)	23.0 (17.0, 28.0)
Missing	2 (0.25%)	–	2 (0.16%)
**Emotional Behavioral Dyscontrol (TBI-QoL)**			
Mean(SD)	52.7 (7.4)	49.8 (8.3)	51.6 (7.9)
Median (IQR)	52.6 (48.2, 57.4)	50.5 (44.1, 55.5)	51.6 (47.0, 57.4)
Missing	71 (9.0%)	60 (12.0%)	131 (10.0%)
**Resilience (TBI-QoL)**			
Mean(SD)	45.8 (9.1)	47.5 (9.5)	46.4 (9.2)
Median (IQR)	45.0 (40.0, 50.3)	47.0 (41.0, 53.9)	46.0 (40.0, 51.5)
Missing	71 (9.0%)	59 (12.0%)	130 (10.0%)
**Alcohol Use (AUDIT-C)**			
Mean(SD)	3.0 (2.4)	2.7 (2.3)	2.9 (2.4)
Median (IQR)	3.0 (1.0, 4.0)	2.0 (1.0, 4.0)	2.0 (1.0, 4.0)
Missing	3 (0.38%)	–	3 (0.23%)
**Drug Use (DAST-10)**			
No Problems	620 (78.8%)	419 (84.3%)	1039 (80.9%)
Low	143 (18.2%)	66 (13.3%)	209 (16.3%)
Moderate to Severe	24 (3.0%)	12 (2.4%)	36 (2.8%)
Missing	1 (0.13%)	1 (0.2%)	2 (0.16%)
**Sleep Quality (PSQI)**			
Mean(SD)	10.6 (4.2)	9.3 (4.3)	10.1 (4.3)
Median (IQR)	11.0 (8.0, 14.0)	9.5 (5.0, 13.0)	10.0 (6.0, 14.0)
Missing	14 (1.8%)	6 (1.2%)	20 (1.6%)
**Neurobehavioral Symptoms (NSI)**			
Mean(SD)	22.9 (14.0)	16.7 (12.9)	20.5 (13.9)
Median (IQR)	21.0 (12.0, 32.0)	14.0 (6.0, 24.0)	18.0 (9.0, 29.0)
Missing	4 (0.51%)	2 (0.4%)	6 (0.47%)

Abbreviations: SD = standard deviation; IQR = interquartile range; mTBI = mild traumatic brain injury; TBI-QoL = Traumatic Brain Injury-Quality of Life; SWLS = Satisfaction with life; NSI = Neurobehavioral Symptom Inventory; PSQI = Pittsburgh Sleep Quality Index; PHQ-9 = Patient Health Questionnaire-9-item; PTSD = Post-Traumatic Stress Disorder; PCL-5 = PTSD Checklist for the Diagnostic and Statistical Manual of Mental Disorders, Fifth Edition; AUDIT-C = Alcohol Use Disorder Identification Test – Consumption; DAST-10 = Drug Abuse Screening Test- 10-item.

*Only among those that had a lifetime history of mTBI in childhood.

### Associations between lifetime childhood mTBI and mental and behavioral health outcomes

After adjustment for demographic, behavioral, and military factors (Fig 1, S1 Table), we observed that compared to the no lifetime mTBI group, Veterans with mTBI in childhood had 36% higher PTSD symptom scores (MR = 1.36, 95% confidence interval (CI): 1.22–1.51, p < 0.001), 8% higher anger scores (MR = 1.08, 95% CI: 1.05–1.10, p < 0.001), 31% higher depression scores (MR = 1.31, 95% CI: 1.17–1.46, p < 0.001), and on average higher anxiety scores (mean difference = 4.3, 95% CI: 2.9-5.6, p < 0.001), emotional-behavioral dyscontrol scores (mean difference = 3.2, 95% CI: 2.2–4.3, p < 0.001), worse sleep quality (mean difference = 1.6, 95% CI: 1.1-2.2, p < 0.001), worse neurobehavioral symptom scores (mean difference = 6.8, 95% CI: 5.2-8.5, p < 0.001), lower satisfaction with life (mean difference = -1.2, 95% CI: -2.1 to -0.27, p = 0.011), and 4% lower resilience scores (MR = 0.96, 95% CI: 0.94–0.99, p = 0.005). There were no significant differences after adjustment for alcohol or drug use scores.

**Fig 1:**
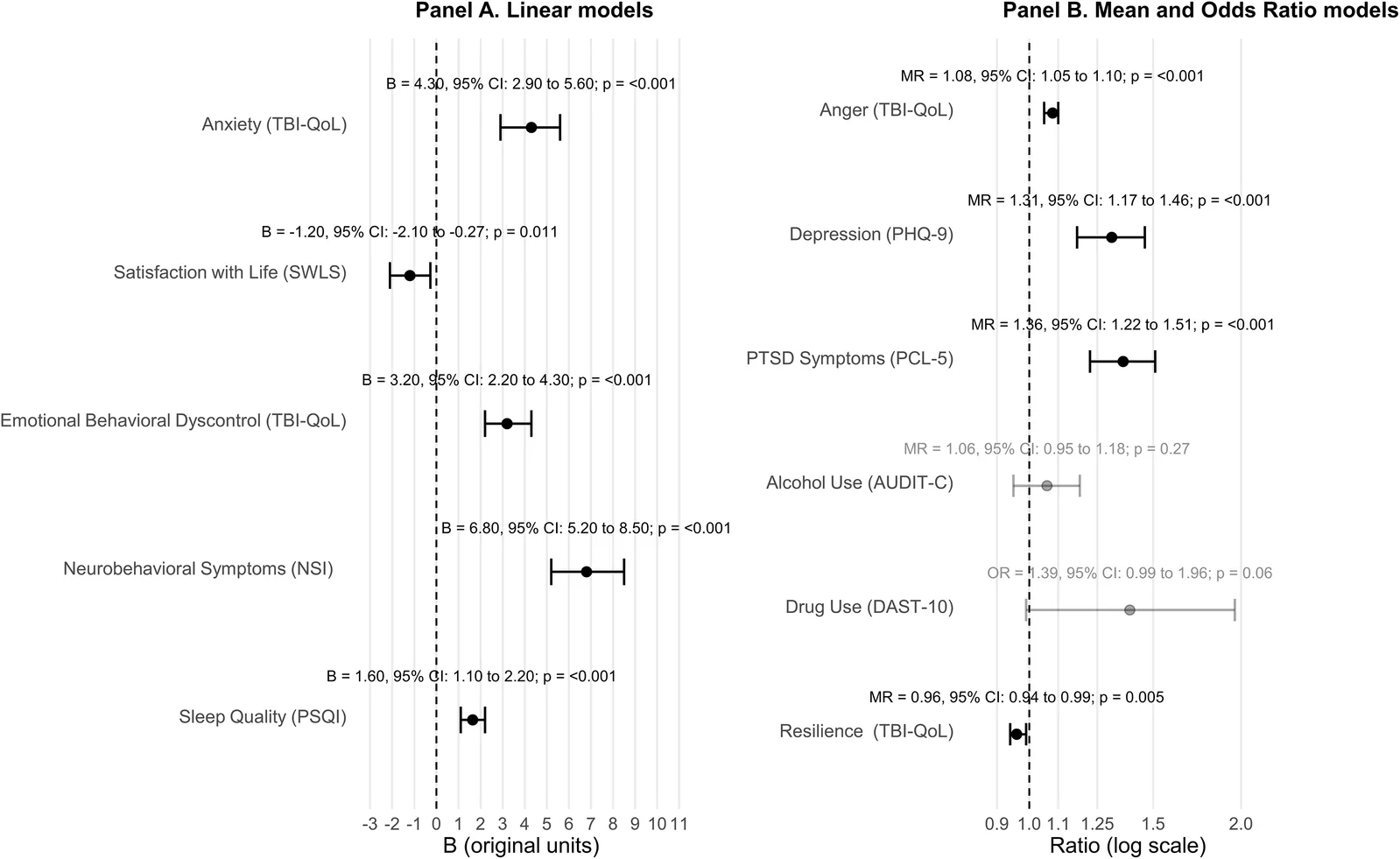
Mental and Behavioral Health Associations with Childhood mTBI History. Mental and behavioral health outcomes presented in Panel A or Panel B depending upon outcome distribution and subsequent model and estimates calculated (Panel A presents linear regression effect estimates; Panel B presents quasi-Poisson, Gamma, and ordinal logistic regression effect estimates). Each outcome is presented with its point effect estimate and 95% confidence interval visually, and with the numeric equivalents and p-values directly above each plot point. All models were adjusted for age, BMI, education, sex, race, tobacco (smoking and chewing dip) use, physical activity, housing status, marital status, and years in the military. Abbreviations: B = beta coefficient; exp(β)= exponentiated beta coefficient; MR = mean ratio; OR = odds ratio; TBI-QoL = Traumatic Brain Injury-Quality of Life; SWLS = Satisfaction with life; NSI = Neurobehavioral Symptom Inventory; PSQI = Pittsburgh Sleep Quality Index; PHQ-9 = Patient Health Questionnaire-9-item; PTSD = Post-Traumatic Stress Disorder; PCL-5 = PTSD Checklist for the Diagnostic and Statistical Manual of Mental Disorders, Fifth Edition; AUDIT-C = Alcohol Use Disorder Identification Test – Consumption; DAST-10 = Drug Abuse Screening Test- 10-item.

### Associations between sociodemographic characteristics and mental and behavioral health outcomes within individuals with lifetime childhood mTBI

After adjustment (Table 2), we observed lower PTSD symptom scores for those with some college education compared to having an education of high school or less (Estimate = -5.7, 95% CI: -10 to -1.1, p = 0.017) and being physically active compared to not being physically active (Estimate = -6.5, 95% CI: -10 to -2.9, p < 0.001). There were higher PTSD symptom scores for those with more than 1 cumulative mTBI (2 mTBIs- Estimate = 7.3, 95% CI: 2.7-12, p = 0.002; 3 or more mTBIs- Estimate = 13, 95% CI: 7.7-18, p < 0.001). Similar patterns were observed for the other mental health outcome variables: Adjusted regression models demonstrated lower anxiety scores for Veterans with some college education and higher anxiety score for sustaining two or more mTBIs; lower anger scores among Veterans who were older, higher educational attainment and physically active and higher anger scores among Veterans who sustained two or more mTBIs; lower depression scores for Veterans who were physically active and higher depression scores for Veterans who are not married, higher BMI, and sustaining two or more mTBIs; lower satisfaction with life for Veterans with less years of age or who are not married and higher satisfaction with life for Veterans who are not White or Black, non-Smokers, physically active, and have greater years of military service. All p’s < 0.05.

**Table 2:** Adjusted Estimates and Odds Ratios (OR) with 95% confidence intervals (CIs) for the association between sociodemographic characteristics and mental and behavioral health outcomes within individuals with lifetime childhood mild traumatic brain injury (mTBI).*.

	Outcomes														
	Mental Health														
	PTSD Symptoms (PCL-5) (N = 591)			Anger (TBI-QoL) (N = 535)			Anxiety (TBI-QoL) (N = 537)			Depression (PHQ-9) (N = 590)			Satisfaction with Life (SWLS) (N = 591)		
Characteristics	Estimate	95% CI	p-value	Estimate	95% CI	p-value	Estimate	95% CI	p-value	Mean Ratio	95% CI	p-value	Estimate	95% CI	p-value
**Sex (Ref = Male)**															
Female	2.1	-2.4, 6.6	0.35	-1.2	-3.8, 1.5	0.39	1.8	-0.87, 4.6	0.18	1.16	0.96, 1.40	0.12	1.1	-0.81, 3.0	0.26
**Age (Years)**	-0.12	-0.31, 0.08	0.25	-0.17	-0.29, -0.05	**0.005**	-0.08	-0.20, 0.05	0.23	0.99	0.98, 1.00	0.14	-0.09	-0.17, 0.00	**0.048**
**Education (Ref = High School or Less)**															
Some College	-5.7	-10, -1.0	**0.017**	-3.3	-6.1, -0.46	**0.023**	-3.5	-6.4, -0.62	**0.018**	0.89	0.74, 1.09	0.27	-0.31	-2.3, 1.7	0.76
Bachelor’s Degree or more	-4.7	-9.5, 0.15	0.058	-3	-5.9, -0.09	**0.043**	-2.7	-5.7, 0.29	0.077	0.94	0.77, 1.16	0.56	0.6	-1.5, 2.7	0.57
**Race (Ref = White)**															
Black	1.9	-2.4, 6.2	0.39	0.14	-2.5, 2.7	0.91	0.46	-2.2, 3.1	0.73	0.91	0.74, 1.10	0.34	0.03	-1.8, 1.9	0.98
Other	-0.52	-5.9, 4.9	0.85	-0.91	-4.2, 2.4	0.59	0.42	-3.0, 3.9	0.81	0.82	0.63, 1.04	0.12	2.3	0.01, 4.6	**0.049**
**Marital Status (Ref = Married)**															
Other	2.9	-0.09, 5.8	0.057	-0.69	-2.5, 1.1	0.45	0.98	-0.86, 2.8	0.3	1.19	1.04, 1.35	**0.01**	-3.6	-4.8, -2.3	**<0.001**
**BMI**	0.08	-0.17, 0.33	0.55	0.11	-0.05, 0.26	0.18	0.06	-0.10, 0.22	0.44	1.01	1.00, 1.02	**0.045**	-0.04	-0.15, 0.07	0.45
**Smoking Status (Ref = Yes)**															
No	-0.02	-3.7, 3.7	0.99	0.79	-1.4, 3.0	0.48	-1.2	-3.5, 1.1	0.31	0.94	0.81, 1.11	0.47	1.7	0.16, 3.3	**0.031**
**Chew Dip Status (Ref = Yes)**															
No	-4.1	-8.8, 0.56	0.084	-0.07	-2.9, 2.7	0.96	-1.3	-4.2, 1.5	0.36	0.9	0.74, 1.09	0.27	0.7	-1.3, 2.7	0.5
**Physical Activity (Ref = No)**															
Yes	-6.5	-10, -2.9	**<0.001**	-2.6	-4.8, -0.45	**0.018**	-0.91	-3.1, 1.3	0.42	0.74	0.64, 0.86	**<0.001**	1.6	0.08, 3.1	**0.039**
**Housing Type (Ref = Private Residence)**															
Other (Group home, College dormitory, Homeless shelter)	6.4	-8.5, 21	0.4	3.4	-6.0, 13	0.48	1.3	-8.5, 11	0.8	1.03	0.53, 1.80	0.92	-4.7	-11, 1.7	0.15
**Years in Military**	-0.06	-0.27, 0.14	0.55	0.05	-0.07, 0.18	0.42	-0.01	-0.14, 0.12	0.87	1	0.99, 1.01	0.6	0.16	0.07, 0.25	**<0.001**
**Time since Last mTBI**	0.16	-0.01, 0.33	0.072	0	-0.11, 0.10	0.97	-0.02	-0.13, 0.09	0.74	1.01	1.00, 1.01	0.1	0.02	-0.05, 0.10	0.53
**Cumulative Lifetime mTBI (Ref = 1)**															
2	7.3	2.7, 12	**0.002**	3.7	0.81, 6.5	**0.012**	4.1	1.1, 7.0	**0.007**	1.4	1.12, 1.75	**0.003**	-0.8	-2.8, 1.2	0.43
3+	13	7.7, 18	**<0.001**	4.9	1.8, 7.9	**0.002**	5	1.8, 8.2	**0.002**	1.73	1.37, 2.19	**<0.001**	-1.2	-3.4, 0.90	0.25

Abbreviations: mTBI = mild traumatic brain injury; TBI-QoL = Traumatic Brain Injury-Quality of Life; SWLS = Satisfaction with life; NSI = Neurobehavioral Symptom Inventory; PSQI = Pittsburgh Sleep Quality Index; PHQ-9 = Patient Health Questionnaire-9-item; PTSD = Post-Traumatic Stress Disorder; PCL-5 = PTSD Checklist for the Diagnostic and Statistical Manual of Mental Disorders, Fifth Edition; AUDIT-C = Alcohol Use Disorder Identification Test – Consumption; DAST-10 = Drug Abuse Screening Test- 10-item; CI = Confidence Interval.

*P-values in bold are significant, p < 0.05.

After adjustment (Table 3), we identified higher emotional-behavioral dyscontrol scores for individuals with higher BMI (Estimate = 0.12, 95% CI: 0.00-0.23, p = 0.047) and three or more mTBIs (Estimate = 2.8, 95% CI: 0.52-5.1, p = 0.017). Similar patterns were observed for the other behavioral health outcome variables: less alcohol use among Veterans who were female, non-smokers, and did not use chew/dip products; lower odds of higher drug use among Veterans with each additional year of military service, who were non-White (Black, Other), and had higher BMI; worse sleep quality (i.e., higher scores) among Veterans who were female or who sustained two or more mTBIs and better sleep quality among Veterans with a Bachelor’s degree or more or who were physically active; lower neurobehavioral symptom scores among Veterans with some college education or a Bachelor’s degree or more and those who were physically active and higher neurobehavioral symptom scores among females, or who sustained two or more mTBIs. All p’s < 0.05.

**Table 3:** Adjusted Estimates and Odds Ratios (OR) with 95% confidence intervals (CIs) for the association between sociodemographic characteristics and mental and behavioral health outcomes within individuals with lifetime childhood mild traumatic brain injury (mTBI).*.

	Outcomes																	
	Behavioral Health																	
	Emotional Behavioral Dyscontrol (TBI-QoL) (N = 537)			Resilience (TBI-QoL) (N = 557)			Alcohol Use (AUDIT-C) (N = 590)			Drug Use (DAST-10) (N = 592)			Sleep Quality (PSQI) (N = 583)			Neuro-Behavioral Symptoms (NSI) (N = 590)		
Characteristics	Estimate	95% CI	p-value	Estimate	95% CI	p-value	Estimate	95% CI	p-value	OR	95% CI	p-value	Estimate	95% CI	p-value	Estimate	95% CI	p-value
**Sex (Ref = Male)**																		
Female	0.23	-1.8, 2.2	0.82	0.5	-1.9, 2.9	0.68	-0.83	-1.5, -0.16	**0.015**	0.75	0.37, 1.44	0.4	1.3	0.18, 2.4	**0.023**	4.8	1.2, 8.3	**0.008**
**Age (Years)**	-0.02	-0.11, 0.07	0.73	-0.05	-0.15, 0.06	0.41	-0.02	-0.05, 0.01	0.11	1.01	0.98, 1.05	0.35	-0.03	-0.08, 0.02	0.3	-0.13	-0.29, 0.03	0.1
**Education (Ref = High School or Less)**																		
Some College	-1.8	-3.9, 0.34	0.1	1.6	-0.94, 4.2	0.21	0.11	-0.59, 0.82	0.75	1.25	0.67, 2.40	0.5	-0.9	-2.1, 0.27	0.13	-4.2	-8.0, -0.46	**0.028**
Bachelor’s Degree or more	-1.7	-3.9, 0.48	0.13	1.7	-0.90, 4.4	0.2	0.35	-0.37, 1.1	0.34	0.66	0.33, 1.31	0.22	-1.2	-2.4, -0.01	**0.048**	-4.8	-8.7, -0.90	**0.016**
**Race (Ref = White)**																		
Black	-1.4	-3.4, 0.55	0.16	2	-0.32, 4.4	0.09	-0.44	-1.1, 0.20	0.18	0.37	0.15, 0.81	**0.018**	0.91	-0.15, 2.0	0.092	-0.67	-4.1, 2.8	0.7
Other	-1.8	-4.3, 0.65	0.15	1.6	-1.4, 4.6	0.3	0.04	-0.77, 0.85	0.92	0.19	0.04, 0.55	**0.007**	-0.38	-1.7, 0.98	0.58	0.44	-3.9, 4.8	0.84
**Marital Status (Ref = Married)**																		
Other	-0.54	-1.9, 0.81	0.43	-1.2	-2.8, 0.41	0.14	0.07	-0.37, 0.51	0.76	1.52	0.99, 2.32	**0.05**	0.07	-0.67, 0.80	0.86	1.1	-1.3, 3.4	0.37
**BMI**	0.12	0.00, 0.23	**0.047**	-0.03	-0.17, 0.11	0.66	0	-0.03, 0.04	0.87	0.94	0.91, 0.98	**0.004**	0.04	-0.03, 0.10	0.27	0.17	-0.03, 0.37	0.093
**Smoking Status (Ref = Yes)**																		
No	0.32	-1.4, 2.0	0.71	1.3	-0.71, 3.3	0.2	-0.8	-1.4, -0.24	**0.005**	0.68	0.42, 1.13	0.13	-0.16	-1.1, 0.76	0.74	0.69	-2.3, 3.6	0.65
**Chew Dip Status (Ref = Yes)**																		
No	-1.2	-3.3, 0.89	0.26	-0.12	-2.7, 2.4	0.92	-0.83	-1.5, -0.13	**0.02**	0.7	0.37, 1.38	0.29	-0.29	-1.4, 0.87	0.62	-0.6	-4.4, 3.2	0.76
**Physical Activity (Ref = No)**																		
Yes	-0.76	-2.4, 0.88	0.36	2.4	0.42, 4.3	**0.018**	0.47	-0.06, 1.0	0.082	1.34	0.78, 2.38	0.3	-1.2	-2.1, -0.36	**0.006**	-5.5	-8.3, -2.6	**<0.001**
**Housing Type (Ref = Private Residence)**																		
Other (Group home, College dormitory, Homeless shelter)	3.7	-3.5, 11	0.31	0.63	-8.0, 9.3	0.89	-1.6	-3.8, 0.65	0.17	0.84	0.04, 6.45	0.88	0.92	-2.7, 4.6	0.62	3.4	-8.5, 15	0.57
**Years in Military**	-0.01	-0.11, 0.08	0.77	0.06	-0.06, 0.17	0.34	0.01	-0.02, 0.04	0.63	0.92	0.89, 0.96	**<0.001**	0.02	-0.03, 0.07	0.38	0.09	-0.07, 0.25	0.28
**Time since Last mTBI**	-0.03	-0.11, 0.05	0.45	0.05	-0.05, 0.14	0.34	-0.01	-0.03, 0.02	0.56	0.99	0.97, 1.02	0.62	0	-0.04, 0.05	0.85	0.08	-0.06, 0.21	0.25
**Cumulative Lifetime mTBI (Ref = 1)**																		
2	1.9	-0.32, 4.0	0.094	-2.2	-4.8, 0.44	0.1	-0.16	-0.86, 0.54	0.65	0.59	0.29, 1.18	0.14	2	0.83, 3.2	**<0.001**	5.8	2.1, 9.5	**0.002**
3+	2.8	0.52, 5.1	**0.017**	-2.3	-5.0, 0.52	0.11	-0.13	-0.88, 0.62	0.74	0.72	0.35, 1.50	0.38	2.3	1.1, 3.6	**<0.001**	11	7.2, 15	**<0.001**

Abbreviations: mTBI = mild traumatic brain injury; TBI-QoL = Traumatic Brain Injury-Quality of Life; SWLS = Satisfaction with life; NSI = Neurobehavioral Symptom Inventory; PSQI = Pittsburgh Sleep Quality Index; PHQ-9 = Patient Health Questionnaire-9-item; PTSD = Post-Traumatic Stress Disorder; PCL-5 = PTSD Checklist for the Diagnostic and Statistical Manual of Mental Disorders, Fifth Edition; AUDIT-C = Alcohol Use Disorder Identification Test – Consumption; DAST-10 = Drug Abuse Screening Test- 10-item; CI = Confidence Interval.

*P-values in bold are significant, p < 0.05.

## Discussion

In this exploratory study, three out of every five (61.3%) Veterans in the study cohort experienced a childhood mTBI during their early lifetime, and their childhood mTBI was associated with worse mental and behavioral health outcomes in select domains. Specifically, Veterans with a childhood mTBI demonstrated worse outcomes on self-reported measures of PTSD symptoms, anger, anxiety, depression, satisfaction with life, emotional-behavioral dyscontrol, resilience, sleep, and neurobehavioral symptoms compared to those without a childhood mTBI. These findings align with previous studies describing the association between mTBI and adverse health outcomes [1–4,22–25,50]. For example, a 2022 comprehensive review by Howlett et al. [50] on the mental health consequences of TBI demonstrated that mTBI contributes to long-term changes in military populations, including new onset of disorders such as PTSD, generalized anxiety disorder, major depressive disorder, and suicidality. Additionally, studies [11–13,51] suggests that mental health disorders can contribute to worse outcomes after mTBI such as prolonged symptom recovery times, chronic disability, unemployment, functional impairments, social isolation, and relationship problems. While our study population was specific to Veterans with combat exposure, future studies can examine if increasing awareness among healthcare providers of mental and behavioral health difficulties for Veterans with a childhood mTBI history may improve screening for these adverse outcomes, as healthcare providers may be better able to identify individuals who would benefit from treatment and support and can provide evidence-based care [52,53]. Importantly, these findings do not establish that childhood mTBI directly caused these later-life outcomes. Alternative explanations are plausible and warrant consideration. For example, childhood mTBI may function as a marker of broader vulnerability rather than a direct cause of later difficulties, as factors such as adverse childhood experiences, socioeconomic disadvantage, family environmental influences, and behavioral traits associated with risk-taking may increase both the likelihood of sustaining a childhood mTBI and the risk of poorer adult mental and behavioral health outcomes [54,55]. Ultimately, childhood mTBI may represent one component within a broader constellation of developmental, psychosocial, and environmental factors that contribute to mental and behavioral health across the lifespan.

Our results also show that among individuals with a history of childhood mTBI, specific domains within mental and behavioral health outcomes were worse in those who sustained multiple childhood mTBIs, with a greater number of cumulative injuries associated with poorer outcomes as reflected by large effect sizes. These results are consistent with numerous other studies that have indicated multiple prior mTBIs are associated with negative health outcomes across the lifespan with a potential additive effect for total mTBI accumulation [56–59]. While much of this evidence comes from athlete populations, additional work in military populations [60–62] has observed similar patterns. Prior research [61] has also found that service members who sustain a mTBI during combat have more negative outcomes than service members who did not sustain a mTBI during combat. This may reflect the circumstances of the injury, which are associated with a higher likelihood of polytrauma and potential for comorbid conditions such as PTSD, pain, headaches [60–62].

In contrast to the pattern observed across several mental and behavioral health domains, we did not observe significant differences in alcohol or drug use scores after adjustment for covariates. This finding may suggest that the association between childhood mTBI and later health outcomes is not uniform across all behavioral domains. Prior literature [63,64] examining the relationship between TBI and substance use has produced mixed findings, with evidence suggesting that substance use may function as both a risk factor for sustaining a TBI and a potential consequence of injury-related neurobehavioral changes, while other studies have emphasized the importance of shared underlying risk factors and confounding influences. Accordingly, the absence of significant associations in our study may reflect the multifactorial nature of substance use behaviors and the influence of factors beyond childhood mTBI history alone. Future longitudinal studies are needed to better clarify the temporal and causal relationships among childhood mTBI, substance use, and other mental and behavioral health outcomes.

Some potential inferences for future studies on protective factors emerged from our findings among Veterans with combat exposure. Notably, Veterans who sustained a childhood mTBI with higher educational attainment or met physical active recommendations demonstrated better mental and behavioral health outcomes in specific domains. While higher education attainment was associated with better outcomes in our study, there have been mixed results on education in the mTBI outcomes literature [65,66], which highlights the complexity of mTBI prognosis. Our finding on physical activity is highly consistent with existing literature in the civilian population where aerobic exercise has demonstrated substantial health benefits and is considered a promising intervention for improving overall health [67,68]. It is also the standard of care for facilitating recovery following mTBI in athletes [69,70]. Furthermore, recent evidence [48] indicates that engagement in moderate-to-vigorous aerobic exercise is associated with improved health-related quality of life among service members and Veterans. However, given the observational nature of this study, these associations should not be interpreted as causal. Alternative explanations, including reverse causation (e.g., individuals with better mental and behavioral health may be more likely to engage in physical activity) and unmeasured factors such as premorbid cognitive ability, socioeconomic advantage, or access to health-promoting resources, may contribute to the observed relationships. Longitudinal analyses are needed to clarify the directionality and causal mechanisms underlying these associations.

Our findings of potential lingering effects of mTBIs experienced in childhood among Veterans with combat exposure highlight the importance of developing interventions aimed at preventing or mitigating long-term sequelae associated with mTBI. Specifically, we observed notable negative effects for mTBIs experienced in childhood (i.e., before military service) that demonstrated lingering effects at time of evaluation. These findings may indicate markers of increased risk that is present before military enlistment, duty, and the numerous exposures experienced warranting unique preventative and treatment strategies. Currently, as noted in one report [71], there is a lack of studies that address prevention or rehabilitation and long-term care among service members and Veterans with mTBI. There is also evidence indicating veterans may face barriers to accessing healthcare, including long wait times at Veterans Health Administration facilities and transportation-related challenges, and may fail to seek, delay, or miss medical care for their TBI [72–75]. This highlights the importance of efforts to improve care (e.g., screening, interventions, rehabilitation services) for veterans to provide unmet needs.

### Limitations

There were several limitations to our study. First, although validated instruments, the mental and behavioral health assessments utilized self-report questionnaires rather than clinical structured interviews. Medical record review for these diagnoses were also not widely feasible. Second, mTBI history in this study and throughout the literature is subject to temporal recall, misclassification, and detection biases related to the medical and layperson recognition of mTBI. For example, there may be recall bias as to when an mTBI occurred, as the average age of Veterans in this study were 43 years old while the average age of first mTBI occurred at 11 years old. Similarly, adult confounders (e.g., education, BMI, physical activity) were used for adjustment, but could potentially introduce overadjustment. Third, the results of this study may be influenced by the sample selection of the cohort and should be interpreted within the context of Veterans with combat exposure. Veterans with combat exposure may differ from the broader Veteran population and civilian populations with respect to trauma burden, military experiences, healthcare utilization, occupational demands, and co-occurring mental health conditions. Consequently, the magnitude and pattern of associations observed in this study may not generalize to all Veterans, Veterans who sustained a mTBI only during adulthood, non-combat Veterans, active-duty service members, or civilian populations with childhood mTBI histories. Future studies should examine these relationships in more diverse military and civilian cohorts to better establish the external validity of these findings. Fourth, early-life confounders (e.g., socioeconomic status, adversity, behavioral traits) were not measured in this study, which could potentially leav residual confounding not accounted for. Fifth, there were a range of outcomes that were examined that differed in measurement scale, recall period, and underlying construct (e.g., symptoms, behaviors, and traits). Thus, these results should be interpreted within each specific domain and not as a single coherent construct for mental and behavioral health. Sixth, this study examined a relatively large number of mental and behavioral health outcomes. Because the analyses were exploratory and intended to evaluate associations across distinct outcome domains, formal adjustment for multiple comparisons was not applied. Consequently, some statistically significant findings may reflect chance associations, and the results should be interpreted as hypothesis-generating pending replication in independent cohorts. Additionally, sex effects were adjusted for in our statistical approaches to better isolate childhood mTBI effects, but this approach is not equivalent to explicitly evaluating sex differences and should not be interpreted as such [76]. Future studies may wish to examine Veterans with mTBI by sex, as prior studies have shown differences in mental and behavioral health outcomes in males and females in the civilian population [2] but studies examining sex differences are more limited in the military population [77]. Lastly, due to the cross-sectional analysis, the associations found in this study do not answer directionality and causality.

## Conclusions

Among a cross-sectional analysis of Veterans with a history of childhood mTBI, mental and behavioral health outcomes in specific domains were worse among those who sustained multiple mTBIs and better among those who were physically active and had higher education. These findings suggest potential targets for future studies to examine pre-enlistment risk factors that may warrant unique screening, preventative, or treatment strategies. For example, future studies can determine if increasing awareness among healthcare providers of mental and behavioral health difficulties for Veterans with a childhood mTBI history can improve screening for these adverse outcomes, and subsequently allow healthcare providers to better identify individuals who would benefit from evidence-based treatment.

## Supporting information

S1 Table Univariate and Multivariable Estimates Between Characteristics and Mental and Behavioral Health Outcomes. Mental and behavioral health outcomes for univariate and multivariable models for PCL-5, TBI-QoL Anger, Anxiety, Resilience and Emotional Behavioral Dyscontrol, PHQ-9, SWLS, AUDIT-C, DAST-10, PSQI, and NSI. Abbreviations: B = beta coefficient; exp(β)= exponentiated beta coefficient; MR = mean ratio; OR = odds ratio; mTBI = mild traumatic brain injury; TBI-QoL = Traumatic Brain Injury-Quality of Life; SWLS = Satisfaction with life; NSI = Neurobehavioral Symptom Inventory; PSQI = Pittsburgh Sleep Quality Index; PHQ-9 = Patient Health Questionnaire-9-item; PTSD = Post-Traumatic Stress Disorder; PCL-5 = PTSD Checklist for the Diagnostic and Statistical Manual of Mental Disorders, Fifth Edition; AUDIT-C = Alcohol Use Disorder Identification Test – Consumption; DAST-10 = Drug Abuse Screening Test- 10-item; CI = Confidence Interval.
(DOCX)

## Notes

Data are available from the National Institutes of Health’s Federal Interagency Traumatic Brain Injury Research Informatics System (FITBIR) at https://fitbir.nih.gov/.
