# Supplementary material for: Association of childhood mild traumatic brain injury with mental and behavioral health outcomes later in life among U.S. Veterans: A LIMBIC-CENC study
Source: PLOS Ment Health. 2026 Oct 21;3(10):e0000679. doi: 10.1371/journal.pmen.0000679 (PMC13686504; doi:10.1371/journal.pmen.0000679)
Supplement: Supplementary file 1 [file pmen.0000679.s001.docx]

S1 Table. Univariate and multivariable associations between characteristics and mental and behavioral health outcomes.*

|  | **Outcomes** | | | | | | | | | | | | | |
| --- | --- | --- | --- | --- | --- | --- | --- | --- | --- | --- | --- | --- | --- | --- |
|  | **Mental Health** | | | | | | | | | | | | | |
|  | **PTSD Symptoms (PCL-5)** | | | | | | | **Anger (TBI-QoL)** | | | | | | |
|  | **Univariate** | | | | **Multivariable**  **(N = 1,034)** | | | **Univariate** | | | | **Multivariable**  **(N = 935)** | | |
| **Characteristic** | **N** | **Mean Ratio** | **95% CI** | **p-value** | **Mean Ratio** | **95% CI** | **p-value** | **N** | **exp (Estimate)** | **95% CI** | **p-value** | **exp (Estimate)** | **95% CI** | **p-value** |
| **mTBI Childhood Group** | 1,284 |  |  | **<0.001** |  |  | **<0.001** | 1,163 |  |  | **<0.001** |  |  | **<0.001** |
| No lifetime mTBI history |  | — | — |  | — | — |  |  | — | — |  | — | — |  |
| Lifetime childhood mTBI |  | 1.28 | 1.17, 1.41 |  | 1.36 | 1.22, 1.51 |  |  | 1.07 | 1.05, 1.09 |  | 1.08 | 1.05, 1.10 |  |
| **Sex** | 1,283 |  |  | **0.041** |  |  | **0.006** | 1,162 |  |  | 0.69 |  |  | 0.43 |
| Male |  | — | — |  | — | — |  |  | — | — |  | — | — |  |
| Female |  | 1.14 | 1.01, 1.28 |  | 1.21 | 1.06, 1.39 |  |  | 0.99 | 0.96, 1.03 |  | 1.01 | 0.98, 1.05 |  |
| **Age** | 1,284 | 0.99 | 0.99, 1.00 | **<0.001** | 1 | 0.99, 1.00 | 0.059 | 1,163 | 1 | 1.00, 1.00 | **<0.001** | 1 | 1.00, 1.00 | **<0.001** |
| **Education** | 1,284 |  |  | **0.001** |  |  | **0.024** | 1,163 |  |  | **<0.001** |  |  | **0.002** |
| High School or Less |  | — | — |  | — | — |  |  | — | — |  | — | — |  |
| Some College |  | 0.86 | 0.74, 0.99 |  | 0.83 | 0.72, 0.97 |  |  | 0.95 | 0.91, 0.98 |  | 0.94 | 0.90, 0.98 |  |
| Bachelor's Degree or more |  | 0.78 | 0.68, 0.89 |  | 0.81 | 0.69, 0.94 |  |  | 0.93 | 0.90, 0.96 |  | 0.93 | 0.89, 0.97 |  |
| **Race** | 1,268 |  |  | **0.008** |  |  | **0.01** | 1,149 |  |  | 0.96 |  |  | 0.83 |
| White |  | — | — |  | — | — |  |  | — | — |  | — | — |  |
| Black |  | 1.2 | 1.06, 1.35 |  | 1.21 | 1.06, 1.37 |  |  | 1 | 0.97, 1.03 |  | 1.01 | 0.97, 1.04 |  |
| Other |  | 1.11 | 0.94, 1.30 |  | 1.14 | 0.96, 1.36 |  |  | 1 | 0.96, 1.05 |  | 1.01 | 0.97, 1.06 |  |
| **Marital Status** | 1,280 |  |  | **0.03** |  |  | 0.95 | 1,159 |  |  | 0.064 |  |  | **0.004** |
| Married |  | — | — |  | — | — |  |  | — | — |  | — | — |  |
| Other |  | 1.1 | 1.01, 1.21 |  | 1 | 0.90, 1.10 |  |  | 0.98 | 0.96, 1.00 |  | 0.96 | 0.94, 0.99 |  |
| **BMI** | 1,271 | 1.01 | 1.00, 1.02 | **0.002** | 1.01 | 1.00, 1.02 | **0.018** | 1,153 | 1 | 1.00, 1.00 | **0.01** | 1 | 1.00, 1.00 | **0.018** |
| **Smoking Status** | 1,284 |  |  | **0.002** |  |  | 0.1 | 1,163 |  |  | **0.026** |  |  | 0.34 |
| Yes |  | — | — |  | — | — |  |  | — | — |  | — | — |  |
| No |  | 0.83 | 0.74, 0.93 |  | 0.9 | 0.79, 1.02 |  |  | 0.97 | 0.94, 1.00 |  | 0.98 | 0.95, 1.02 |  |
| **Chew Dip Status** | 1,279 |  |  | 0.06 |  |  | 0.06 | 1,159 |  |  | 0.15 |  |  | 0.58 |
| Yes |  | — | — |  | — | — |  |  | — | — |  | — | — |  |
| No |  | 0.85 | 0.73, 1.01 |  | 0.84 | 0.71, 1.01 |  |  | 0.97 | 0.93, 1.01 |  | 0.99 | 0.94, 1.04 |  |
| **Physical Activity** | 1,278 |  |  | **<0.001** |  |  | **<0.001** | 1,159 |  |  | **0.002** |  |  | 0.11 |
| No |  | — | — |  | — | — |  |  | — | — |  | — | — |  |
| Yes |  | 0.75 | 0.68, 0.84 |  | 0.82 | 0.73, 0.92 |  |  | 0.96 | 0.93, 0.98 |  | 0.97 | 0.94, 1.01 |  |
| **Housing Type** | 1,278 |  |  | 0.21 |  |  | 0.34 | 1,158 |  |  | 0.23 |  |  | 0.3 |
| Private residence |  | — | — |  | — | — |  |  | — | — |  | — | — |  |
| Other (Group home, College dormitory, Homeless shelter) |  | 1.36 | 0.83, 2.08 |  | 1.28 | 0.76, 1.99 |  |  | 1.08 | 0.95, 1.24 |  | 1.08 | 0.94, 1.26 |  |
| **Years in Military** | 1,043 | 0.99 | 0.99, 1.00 | **0.004** | 1 | 0.99, 1.01 | 0.68 | 940 | 1 | 1.00, 1.00 | **0.013** | 1 | 1.00, 1.00 | 0.31 |
| Abbreviations: mTBI = mild traumatic brain injury; TBI-QoL = Traumatic Brain Injury-Quality of Life; SWLS= Satisfaction with life; NSI = Neurobehavioral Symptom Inventory; PSQI = Pittsburgh Sleep Quality Index; PHQ-9= Patient Health Questionnaire-9-item; PTSD = Post-Traumatic Stress Disorder; PCL-5= PTSD Checklist for the Diagnostic and Statistical Manual of Mental Disorders, Fifth Edition; AUDIT-C= Alcohol Use Disorder Identification Test – Consumption; DAST-10= Drug Abuse Screening Test- 10-item; CI = Confidence Interval. | | | | | | | | | | | | | | |
| *P-values in bold are significant, p < 0.05. | | | | | | | | | | | | | | |

| S1 Table [Continued]. Univariate and multivariable associations between characteristics and mental and behavioral health outcomes.* | | | | | | | | | | | | | | |
| --- | --- | --- | --- | --- | --- | --- | --- | --- | --- | --- | --- | --- | --- | --- |
|  | **Outcomes** | | | | | | | | | | | | | |
|  | **Mental Health** | | | | | | | | | | | | | |
|  | **Anxiety (TBI-QoL)** | | | | | | | **Depression (PHQ-9)** | | | | | | |
|  | **Univariate** | | | | **Multivariable**  **(N = 937)** | | | **Univariate** | | | | **Multivariable**  **(N = 1,030)** | | |
| **Characteristic** | **N** | **Estimate** | **95% CI** | **p-value** | **Estimate** | **95% CI** | **p-value** | **N** | **Mean Ratio** | **95% CI** | **p-value** | **Mean Ratio** | **95% CI** | **p-value** |
| **mTBI Childhood Group** | 1,163 |  |  | **<0.001** |  |  | **<0.001** | 1,278 |  |  | **<0.001** |  |  | **<0.001** |
| No lifetime mTBI history |  | — | — |  | — | — |  |  | — | — |  | — | — |  |
| Lifetime childhood mTBI |  | 3.9 | 2.7, 5.1 |  | 4.3 | 2.9, 5.6 |  |  | 1.28 | 1.16, 1.41 |  | 1.31 | 1.17, 1.46 |  |
| **Sex** | 1,162 |  |  | 0.1 |  |  | **0.011** | 1,277 |  |  | **0.041** |  |  | **0.004** |
| Male |  | — | — |  | — | — |  |  | — | — |  | — | — |  |
| Female |  | 1.4 | -0.25, 3.1 |  | 2.4 | 0.55, 4.2 |  |  | 1.14 | 1.01, 1.29 |  | 1.23 | 1.07, 1.41 |  |
| **Age** | 1,163 | -0.12 | -0.17, -0.06 | **<0.001** | -0.09 | -0.17, -0.02 | **0.012** | 1,278 | 0.99 | 0.99, 1.0 | **<0.001** | 0.99 | 0.99, 1.00 | **0.005** |
| **Education** | 1,163 |  |  | **0.008** |  |  | **0.01** | 1,278 |  |  | **0.002** |  |  | 0.19 |
| High School or Less |  | — | — |  | — | — |  |  | — | — |  | — | — |  |
| Some College |  | -2.4 | -4.4, -0.38 |  | -3.1 | -5.2, -0.93 |  |  | 0.84 | 0.73, 0.98 |  | 0.88 | 0.75, 1.03 |  |
| Bachelor's Degree or more |  | -3.1 | -5.1, -1.1 |  | -3.2 | -5.4, -1.1 |  |  | 0.77 | 0.67, 0.89 |  | 0.86 | 0.73, 1.01 |  |
| **Race** | 1,149 |  |  | 0.98 |  |  | 0.67 | 1,262 |  |  | 0.74 |  |  | 0.64 |
| White |  | — | — |  | — | — |  |  | — | — |  | — | — |  |
| Black |  | -0.01 | -1.7, 1.6 |  | 0.61 | -1.2, 2.4 |  |  | 1.05 | 0.92, 1.19 |  | 1.06 | 0.92, 1.22 |  |
| Other |  | 0.25 | -2.0, 2.5 |  | 0.87 | -1.6, 3.3 |  |  | 1 | 0.83, 1.19 |  | 1.06 | 0.87, 1.27 |  |
| **Marital Status** | 1,159 |  |  | 0.18 |  |  | 0.94 | 1,274 |  |  | **<0.001** |  |  | 0.13 |
| Married |  | — | — |  | — | — |  |  | — | — |  | — | — |  |
| Other |  | 0.83 | -0.38, 2.1 |  | -0.05 | -1.4, 1.3 |  |  | 1.17 | 1.07, 1.28 |  | 1.08 | 0.98, 1.20 |  |
| **BMI** | 1,154 | 0.13 | 0.01, 0.24 | **0.027** | 0.13 | 0.01, 0.26 | **0.03** | 1,265 | 1.02 | 1.01, 1.03 | **<0.001** | 1.02 | 1.01, 1.03 | **<0.001** |
| **Smoking Status** | 1,163 |  |  | **<0.001** |  |  | **0.047** | 1,278 |  |  | **0.018** |  |  | 0.19 |
| Yes |  | — | — |  | — | — |  |  | — | — |  | — | — |  |
| No |  | -2.9 | -4.5, -1.3 |  | -1.8 | -3.6, -0.02 |  |  | 0.86 | 0.76, 0.97 |  | 0.91 | 0.80, 1.04 |  |
| **Chew Dip Status** | 1,159 |  |  | **0.025** |  |  | 0.061 | 1,274 |  |  | **0.03** |  |  | 0.085 |
| Yes |  | — | — |  | — | — |  |  | — | — |  | — | — |  |
| No |  | -2.6 | -4.8, -0.32 |  | -2.5 | -5.2, 0.12 |  |  | 0.83 | 0.70, 0.98 |  | 0.85 | 0.71, 1.02 |  |
| **Physical Activity** | 1,159 |  |  | **0.013** |  |  | 0.14 | 1,273 |  |  | **<0.001** |  |  | **<0.001** |
| No |  | — | — |  | — | — |  |  | — | — |  | — | — |  |
| Yes |  | -2 | -3.6, -0.42 |  | -1.3 | -3.0, 0.42 |  |  | 0.7 | 0.63, 0.79 |  | 0.75 | 0.67, 0.85 |  |
| **Housing Type** | 1,158 |  |  | 0.41 |  |  | 0.74 | 1,272 |  |  | 0.95 |  |  | 0.74 |
| Private residence |  | — | — |  | — | — |  |  | — | — |  | — | — |  |
| Other (Group home, College dormitory, Homeless shelter) |  | 3 | -4.2, 10 |  | 1.4 | -6.7, 9.4 |  |  | 0.98 | 0.53, 1.64 |  | 0.91 | 0.47, 1.55 |  |
| **Years in Military** | 941 | -0.1 | -0.17, -0.02 | **0.009** | 0.01 | -0.08, 0.11 | 0.81 | 1,039 | 0.99 | 0.99, 1.00 | **0.003** | 1 | 0.99, 1.01 | 0.69 |

| S1 Table [Continued]. Univariate and multivariable associations between characteristics and mental and behavioral health outcomes.* | | | | | | | | | | | | | | | | |
| --- | --- | --- | --- | --- | --- | --- | --- | --- | --- | --- | --- | --- | --- | --- | --- | --- |
|  | **Outcomes** | | | | | | | | | | | | | | | |
|  | **Mental Health** | | | | | | | | | **Behavioral Health** | | | | | | |
|  | **Satisfaction with Life (SWLS)**  **(N = 1,034)** | | | | | | | | | **Emotional Behavioral Dyscontrol (TBI-QoL) (N = 930)** | | | | | | |
|  | **Univariate** | | | | | **Multivariable** | | | | **Univariate** | | | | **Multivariable** | | |
| **Characteristic** | **N** | **Estimate** | **95% CI** | **p-value** | **Estimate** | | **95% CI** | **p-value** | **N** | | **Estimate** | **95% CI** | **p-value** | **Estimate** | **95% CI** | **p-value** |
| **mTBI Childhood Group** | 1,284 |  |  | 0.067 |  | |  | **0.011** | 1,155 | |  |  | **<0.001** |  |  | **<0.001** |
| No lifetime mTBI history |  | — | — |  | — | | — |  |  | | — | — |  | — | — |  |
| Lifetime childhood mTBI |  | -0.79 | -1.6, 0.06 |  | -1.2 | | -2.1, -0.27 |  |  | | 2.9 | 2.0, 3.9 |  | 3.2 | 2.2, 4.3 |  |
| **Sex** | 1,283 |  |  | 0.82 |  | |  | 0.84 | 1,154 | |  |  | 0.61 |  |  | 0.056 |
| Male |  | — | — |  | — | | — |  |  | | — | — |  | — | — |  |
| Female |  | -0.13 | -1.3, 1.0 |  | 0.13 | | -1.1, 1.4 |  |  | | 0.34 | -0.95, 1.6 |  | 1.4 | -0.04, 2.8 |  |
| **Age** | 1,284 | 0.07 | 0.03, 0.11 | **<0.001** | 0 | | -0.04, 0.05 | 0.86 | 1,155 | | -0.06 | -0.10, -0.01 | **0.008** | -0.06 | -0.12, -0.01 | **0.032** |
| **Education** | 1,284 |  |  | **<0.001** |  | |  | **0.021** | 1,155 | |  |  | **0.007** |  |  | **0.022** |
| High School or Less |  | — | — |  | — | | — |  |  | | — | — |  | — | — |  |
| Some College |  | -0.08 | -1.5, 1.3 |  | -0.09 | | -1.6, 1.4 |  |  | | -1.7 | -3.3, -0.19 |  | -2 | -3.6, -0.31 |  |
| Bachelor's Degree or more |  | 2.6 | 1.2, 4.0 |  | 1.3 | | -0.23, 2.7 |  |  | | -2.4 | -3.9, -0.90 |  | -2.4 | -4.0, -0.68 |  |
| **Race** | 1,268 |  |  | **0.015** |  | |  | 0.18 | 1,141 | |  |  | 0.12 |  |  | 0.38 |
| White |  | — | — |  | — | | — |  |  | | — | — |  | — | — |  |
| Black |  | -1.6 | -2.8, -0.46 |  | -0.98 | | -2.2, 0.23 |  |  | | -1.3 | -2.6, -0.06 |  | -0.97 | -2.4, 0.42 |  |
| Other |  | 0.48 | -1.1, 2.0 |  | 0.61 | | -1.0, 2.3 |  |  | | -0.04 | -1.8, 1.7 |  | 0.02 | -1.9, 1.9 |  |
| **Marital Status** | 1,280 |  |  | **<0.001** |  | |  | **<0.001** | 1,151 | |  |  | 0.062 |  |  | **0.031** |
| Married |  | — | — |  | — | | — |  |  | | — | — |  | — | — |  |
| Other |  | -4.1 | -4.9, -3.3 |  | -3 | | -3.9, -2.1 |  |  | | -0.89 | -1.8, 0.04 |  | -1.2 | -2.2, -0.11 |  |
| **BMI** | 1,271 | -0.12 | -0.20, -0.04 | **0.003** | -0.12 | | -0.20, -0.03 | **0.005** | 1,145 | | 0.16 | 0.07, 0.25 | **<0.001** | 0.16 | 0.06, 0.25 | **0.001** |
| **Smoking Status** | 1,284 |  |  | **<0.001** |  | |  | **<0.001** | 1,155 | |  |  | 0.089 |  |  | 0.66 |
| Yes |  | — | — |  | — | | — |  |  | | — | — |  | — | — |  |
| No |  | 3.5 | 2.4, 4.7 |  | 2.2 | | 0.97, 3.4 |  |  | | -1.1 | -2.4, 0.17 |  | -0.32 | -1.7, 1.1 |  |
| **Chew Dip Status** | 1,279 |  |  | 0.35 |  | |  | 0.37 | 1,151 | |  |  | **0.006** |  |  | 0.12 |
| Yes |  | — | — |  | — | | — |  |  | | — | — |  | — | — |  |
| No |  | 0.77 | -0.84, 2.4 |  | 0.83 | | -1.0, 2.7 |  |  | | -2.4 | -4.2, -0.71 |  | -1.6 | -3.7, 0.45 |  |
| **Physical Activity** | 1,278 |  |  | **<0.001** |  | |  | **0.017** | 1,151 | |  |  | **0.024** |  |  | 0.41 |
| No |  | — | — |  | — | | — |  |  | | — | — |  | — | — |  |
| Yes |  | 2.8 | 1.7, 3.9 |  | 1.4 | | 0.25, 2.5 |  |  | | -1.4 | -2.6, -0.19 |  | -0.55 | -1.9, 0.76 |  |
| **Housing Type** | 1,278 |  |  | **0.049** |  | |  | 0.17 | 1,150 | |  |  | 0.31 |  |  | 0.26 |
| Private residence |  | — | — |  | — | | — |  |  | | — | — |  | — | — |  |
| Other (Group home, College dormitory, Homeless shelter) |  | -5 | -9.9, -0.03 |  | -3.7 | | -8.9, 1.6 |  |  | | 2.8 | -2.7, 8.3 |  | 3.5 | -2.6, 9.7 |  |
| **Years in Military** | 1,043 | 0.16 | 0.11, 0.21 | **<0.001** | 0.09 | | 0.02, 0.15 | **0.006** | 935 | | -0.04 | -0.09, 0.02 | 0.18 | 0.02 | -0.05, 0.09 | 0.56 |

| [CONTINUED] S1 Table. Univariate and multivariable associations between characteristics and mental and behavioral health outcomes.* | | | | | | | | | | | | | | | |
| --- | --- | --- | --- | --- | --- | --- | --- | --- | --- | --- | --- | --- | --- | --- | --- |
|  | **Outcomes** | | | | | | | | | | | | | | |
|  | **Behavioral Health** | | | | | | | | | | | | | | |
|  | **Resilience (TBI-QoL)** | | | | | | | **Alcohol Use (AUDIT-C)** | | | | | | | |
|  | **Univariate** | | | | **Multivariable**  **(N = 932)** | | | **Univariate** | | | | | **Multivariable**  **(N = 1,032)** | | |
| **Characteristic** | **N** | **exp (Estimate)** | **95% CI** | **p-value** | **exp (Estimate)** | **95% CI** | **p-value** | **N** | **Mean Ratio** | **95% CI** | **p-value** | **Mean Ratio** | | **95% CI** | **p-value** |
| **mTBI Childhood Group** | 1,156 |  |  | **0.003** |  |  | **0.005** | 1,283 |  |  | **0.005** |  | |  | 0.27 |
| No lifetime mTBI history |  | — | — |  | — | — |  |  | — | — |  | — | | — |  |
| Lifetime childhood mTBI |  | 0.97 | 0.94, 0.99 |  | 0.96 | 0.94, 0.99 |  |  | 1.14 | 1.04, 1.25 |  | 1.06 | | 0.95, 1.18 |  |
| **Sex** | 1,155 |  |  | 0.26 |  |  | 0.21 | 1,282 |  |  | **<0.001** |  | |  | **<0.001** |
| Male |  | — | — |  | — | — |  |  | — | — |  | — | | — |  |
| Female |  | 0.98 | 0.95, 1.01 |  | 0.98 | 0.94, 1.01 |  |  | 0.68 | 0.59, 0.79 |  | 0.72 | | 0.61, 0.84 |  |
| **Age** | 1,156 | 1 | 1.00, 1.00 | **0.002** | 1 | 1.00, 1.00 | 0.16 | 1,283 | 0.99 | 0.99, 1.00 | **<0.001** | 0.99 | | 0.99, 1.00 | **0.004** |
| **Education** | 1,156 |  |  | **0.002** |  |  | 0.24 | 1,283 |  |  | 0.85 |  | |  | 0.31 |
| High School or Less |  | — | — |  | — | — |  |  | — | — |  | — | | — |  |
| Some College |  | 1.04 | 1.00, 1.08 |  | 1.03 | 0.99, 1.08 |  |  | 0.96 | 0.82, 1.12 |  | 1.04 | | 0.88, 1.24 |  |
| Bachelor's Degree or more |  | 1.07 | 1.03, 1.11 |  | 1.04 | 0.99, 1.08 |  |  | 0.96 | 0.83, 1.11 |  | 1.12 | | 0.94, 1.34 |  |
| **Race** | 1,142 |  |  | 0.59 |  |  | 0.4 | 1,268 |  |  | **0.027** |  | |  | 0.17 |
| White |  | — | — |  | — | — |  |  | — | — |  | — | | — |  |
| Black |  | 1.02 | 0.98, 1.05 |  | 1.02 | 0.99, 1.06 |  |  | 0.84 | 0.74, 0.96 |  | 0.89 | | 0.76, 1.03 |  |
| Other |  | 1.01 | 0.97, 1.05 |  | 1.01 | 0.97, 1.06 |  |  | 1.02 | 0.86, 1.20 |  | 1.08 | | 0.89, 1.29 |  |
| **Marital Status** | 1,152 |  |  | **0.017** |  |  | 0.15 | 1,279 |  |  | 0.8 |  | |  | 0.47 |
| Married |  | — | — |  | — | — |  |  | — | — |  | — | | — |  |
| Other |  | 0.97 | 0.95, 1.0 |  | 0.98 | 0.95, 1.01 |  |  | 1.01 | 0.92, 1.11 |  | 1.04 | | 0.93, 1.16 |  |
| **BMI** | 1,146 | 1 | 1.00, 1.00 | **0.021** | 1 | 1.00, 1.00 | **0.034** | 1,270 | 1 | 0.99, 1.01 | 0.48 | 1 | | 0.99, 1.01 | 0.92 |
| **Smoking Status** | 1,156 |  |  | **<0.001** |  |  | **0.01** | 1,283 |  |  | **<0.001** |  | |  | **0.003** |
| Yes |  | — | — |  | — | — |  |  | — | — |  | — | | — |  |
| No |  | 1.07 | 1.03, 1.10 |  | 1.05 | 1.01, 1.08 |  |  | 0.8 | 0.71, 0.89 |  | 0.81 | | 0.71, 0.93 |  |
| **Chew Dip Status** | 1,152 |  |  | 0.37 |  |  | 0.61 | 1,278 |  |  | **<0.001** |  | |  | **0.029** |
| Yes |  | — | — |  | — | — |  |  | — | — |  | — | | — |  |
| No |  | 1.02 | 0.98, 1.07 |  | 1.01 | 0.96, 1.07 |  |  | 0.74 | 0.63, 0.86 |  | 0.81 | | 0.67, 0.98 |  |
| **Physical Activity** | 1,152 |  |  | **<0.001** |  |  | **0.004** | 1,277 |  |  | **0.035** |  | |  | **0.012** |
| No |  | — | — |  | — | — |  |  | — | — |  | — | | — |  |
| Yes |  | 1.06 | 1.03, 1.10 |  | 1.05 | 1.02, 1.09 |  |  | 1.14 | 1.01, 1.30 |  | 1.19 | | 1.04, 1.38 |  |
| **Housing Type** | 1,151 |  |  | 0.9 |  |  | 0.89 | 1,277 |  |  | 0.091 |  | |  | 0.059 |
| Private residence |  | — | — |  | — | — |  |  | — | — |  | — | | — |  |
| Other (Group home, College dormitory, Homeless shelter) |  | 0.99 | 0.87, 1.14 |  | 1.01 | 0.87, 1.19 |  |  | 0.58 | 0.26, 1.08 |  | 0.49 | | 0.19, 1.02 |  |
| **Years in Military** | 937 | 1 | 1.00, 1.00 | **0.037** | 1 | 1.00, 1.00 | 0.95 | 1,041 | 1 | 0.99, 1.00 | 0.16 | 1 | | 1.00, 1.01 | 0.4 |

| [CONTINUED] S1 Table. Univariate and multivariable associations between characteristics and mental and behavioral health outcomes.* | | | | | | | | | | | | | | |
| --- | --- | --- | --- | --- | --- | --- | --- | --- | --- | --- | --- | --- | --- | --- |
|  | **Outcomes** | | | | | | | | | | | | | |
|  | **Behavioral Health** | | | | | | | | | | | | | |
|  | **Drug Use (DAST-10)**  **(N = 1,034)** | | | | | | | **Sleep Quality (PSQI) (N = 1,020)** | | | | | | |
|  | **Univariate** | | | | **Multivariable** | | | **Univariate** | | | | **Multivariable** | | |
| **Characteristic** | **N** | **OR** | **95% CI** | **p-value** | **OR** | **95% CI** | **p-value** | **N** | **Estimate** | **95% CI** | **p-value** | **Estimate** | **95% CI** | **p-value** |
| **mTBI Childhood Group** | 1,284 |  |  | **0.014** |  |  | 0.06 | 1,266 |  |  | **<0.001** |  |  | **<0.001** |
| No lifetime mTBI history |  | — | — |  | — | — |  |  | — | — |  | — | — |  |
| Lifetime childhood mTBI |  | 1.44 | 1.08, 1.94 |  | 1.39 | 0.99, 1.96 |  |  | 1.3 | 0.83, 1.8 |  | 1.6 | 1.1, 2.2 |  |
| **Sex** | 1,283 |  |  | 0.071 |  |  | 0.2 | 1,265 |  |  | **0.018** |  |  | **<0.001** |
| Male |  | — | — |  | — | — |  |  | — | — |  | — | — |  |
| Female |  | 0.68 | 0.43, 1.03 |  | 0.73 | 0.45, 1.17 |  |  | 0.81 | 0.14, 1.5 |  | 1.3 | 0.56, 2.1 |  |
| **Age** | 1,284 | 0.97 | 0.95, 0.98 | **<0.001** | 1 | 0.98, 1.02 | 0.93 | 1,266 | -0.03 | -0.05, -0.01 | **0.005** | -0.02 | -0.05, 0.01 | 0.14 |
| **Education** | 1,284 |  |  | **<0.001** |  |  | 0.15 | 1,266 |  |  | **<0.001** |  |  | **0.01** |
| High School or Less |  | — | — |  | — | — |  |  | — | — |  | — | — |  |
| Some College |  | 1.11 | 0.72, 1.76 |  | 1.36 | 0.83, 2.29 |  |  | -0.68 | -1.5, 0.14 |  | -0.82 | -1.7, 0.06 |  |
| Bachelor's Degree or more |  | 0.55 | 0.35, 0.88 |  | 0.99 | 0.59, 1.69 |  |  | -1.4 | -2.2, -0.60 |  | -1.3 | -2.2, -0.45 |  |
| **Race** | 1,268 |  |  | **0.006** |  |  | **0.015** | 1,251 |  |  | **<0.001** |  |  | **<0.001** |
| White |  | — | — |  | — | — |  |  | — | — |  | — | — |  |
| Black |  | 0.68 | 0.44, 1.02 |  | 0.75 | 0.46, 1.20 |  |  | 1.3 | 0.64, 2.0 |  | 1.6 | 0.91, 2.4 |  |
| Other |  | 0.42 | 0.20, 0.79 |  | 0.38 | 0.17, 0.77 |  |  | 0.52 | -0.37, 1.4 |  | 0.9 | -0.08, 1.9 |  |
| **Marital Status** | 1,280 |  |  | **<0.001** |  |  | 0.13 | 1,262 |  |  | **0.037** |  |  | 0.9 |
| Married |  | — | — |  | — | — |  |  | — | — |  | — | — |  |
| Other |  | 1.89 | 1.43, 2.51 |  | 1.29 | 0.93, 1.79 |  |  | 0.51 | 0.03, 1.0 |  | 0.03 | -0.52, 0.59 |  |
| **BMI** | 1,271 | 0.96 | 0.94, 0.99 | **0.006** | 0.97 | 0.94, 1.0 | **0.02** | 1,254 | 0.08 | 0.03, 0.12 | **<0.001** | 0.06 | 0.01, 0.10 | **0.024** |
| **Smoking Status** | 1,284 |  |  | **<0.001** |  |  | **<0.001** | 1,266 |  |  | 0.11 |  |  | 0.47 |
| Yes |  | — | — |  | — | — |  |  | — | — |  | — | — |  |
| No |  | 0.39 | 0.28, 0.55 |  | 0.51 | 0.35, 0.76 |  |  | -0.54 | -1.2, 0.12 |  | -0.26 | -0.99, 0.46 |  |
| **Chew Dip Status** | 1,279 |  |  | **0.038** |  |  | 0.2 | 1,261 |  |  | 0.44 |  |  | 0.33 |
| Yes |  | — | — |  | — | — |  |  | — | — |  | — | — |  |
| No |  | 0.59 | 0.37, 0.97 |  | 0.67 | 0.38, 1.24 |  |  | -0.37 | -1.3, 0.56 |  | -0.53 | -1.6, 0.55 |  |
| **Physical Activity** | 1,278 |  |  | 0.83 |  |  | 0.16 | 1,260 |  |  | **<0.001** |  |  | **0.014** |
| No |  | — | — |  | — | — |  |  | — | — |  | — | — |  |
| Yes |  | 0.96 | 0.67, 1.40 |  | 1.35 | 0.89, 2.10 |  |  | -1.2 | -1.9, -0.61 |  | -0.86 | -1.5, -0.18 |  |
| **Housing Type** | 1,278 |  |  | 0.12 |  |  | 0.71 | 1,260 |  |  | 0.16 |  |  | 0.4 |
| Private residence |  | — | — |  | — | — |  |  | — | — |  | — | — |  |
| Other (Group home, College dormitory, Homeless shelter) |  | 3.58 | 0.70, 15.6 |  | 1.4 | 0.19, 7.41 |  |  | 2 | -0.82, 4.8 |  | 1.3 | -1.8, 4.5 |  |
| **Years in Military** | 1,043 | 0.92 | 0.90, 0.94 | **<0.001** | 0.93 | 0.90, 0.95 | **<0.001** | 1,028 | -0.01 | -0.04, 0.01 | 0.32 | 0.02 | -0.02, 0.05 | 0.39 |

[CONTINUED] S1 Table. Univariate and multivariable associations between characteristics and mental and behavioral health outcomes.*

|  | **Outcomes** | | | | | | | | |
| --- | --- | --- | --- | --- | --- | --- | --- | --- | --- |
|  | **Behavioral Health** | | | | | | | | |
|  | **Neuro-Behavioral Symptoms (NSI)** | | | | | | | | |
|  | **Univariate** | | | | | **Multivariable**  **(N = 1,032)** | | | |
| **Characteristic** | **N** | **Estimate** | **95% CI** | **p-value** | **Estimate** | | **95% CI** | **p-value** |  |
| **mTBI Childhood Group** | 1,280 |  |  | **<0.001** |  | |  | **<0.001** |  |
| No lifetime mTBI history |  | — | — |  | — | | — |  |  |
| Lifetime childhood mTBI |  | 6.2 | 4.7, 7.7 |  | 6.8 | | 5.2, 8.5 |  |  |
| **Sex** | 1,279 |  |  | **0.002** |  | |  | **<0.001** |  |
| Male |  | — | — |  | — | | — |  |  |
| Female |  | 3.4 | 1.2, 5.5 |  | 5.5 | | 3.2, 7.8 |  |  |
| **Age** | 1,280 | -0.12 | -0.19, -0.05 | **0.001** | -0.1 | | -0.19, -0.01 | **0.024** |  |
| **Education** | 1,280 |  |  | **0.002** |  | |  | **0.002** |  |
| High School or Less |  | — | — |  | — | | — |  |  |
| Some College |  | -2.8 | -5.4, -0.12 |  | -3.3 | | -6.1, -0.62 |  |  |
| Bachelor's Degree or more |  | -4.4 | -6.9, -1.8 |  | -4.8 | | -7.6, -2.1 |  |  |
| **Race** | 1,264 |  |  | 0.35 |  | |  | 0.15 |  |
| White |  | — | — |  | — | | — |  |  |
| Black |  | 1.3 | -0.82, 3.4 |  | 1.5 | | -0.70, 3.8 |  |  |
| Other |  | 1.4 | -1.5, 4.3 |  | 2.5 | | -0.55, 5.5 |  |  |
| **Marital Status** | 1,276 |  |  | 0.42 |  | |  | 0.73 |  |
| Married |  | — | — |  | — | | — |  |  |
| Other |  | 0.63 | -0.91, 2.2 |  | -0.3 | | -2.0, 1.4 |  |  |
| **BMI** | 1,268 | 0.31 | 0.17, 0.46 | **<0.001** | 0.27 | | 0.12, 0.42 | **<0.001** |  |
| **Smoking Status** | 1,280 |  |  | 0.35 |  | |  | 0.96 |  |
| Yes |  | — | — |  | — | | — |  |  |
| No |  | -1 | -3.1, 1.1 |  | -0.06 | | -2.3, 2.2 |  |  |
| **Chew Dip Status** | 1,275 |  |  | 0.17 |  | |  | 0.28 |  |
| Yes |  | — | — |  | — | | — |  |  |
| No |  | -2.1 | -5.0, 0.90 |  | -1.9 | | -5.2, 1.5 |  |  |
| **Physical Activity** | 1,274 |  |  | **<0.001** |  | |  | **<0.001** |  |
| No |  | — | — |  | — | | — |  |  |
| Yes |  | -4.4 | -6.4, -2.4 |  | -3.9 | | -6.0, -1.8 |  |  |
| **Housing Type** | 1,274 |  |  | 0.44 |  | |  | 0.52 |  |
| Private residence |  | — | — |  | — | | — |  |  |
| Other (Group home, College dormitory, Homeless shelter) |  | 3.6 | -5.5, 13 |  | 3.2 | | -6.5, 13 |  |  |
| **Years in Military** | 1,040 | -0.08 | -0.17, 0.01 | 0.093 | 0.06 | | -0.05, 0.17 | 0.29 |  |
