# Supplementary material for: Association of childhood mild traumatic brain injury with mental and behavioral health outcomes later in life among U.S. Veterans: A LIMBIC-CENC study
Source: PLOS Ment Health. 2026 Oct 21;3(10):e0000679. doi: 10.1371/journal.pmen.0000679 (PMC13686504; doi:10.1371/journal.pmen.0000679)
Supplement: Supplementary file 2 [file pmen.0000679.s003.docx]

Journal Requirements:

1. Please provide a detailed online Financial Disclosure statement. This is published with the article. It must therefore be completed in full sentences and contain the exact wording you wish to be published.
We have now completed and uploaded the financial disclosure statement.

a) Please clarify all sources of financial support for your study. List the grants, grant numbers, and organizations that funded your study, including funding received from your institution. Please note that suppliers of material support, including research materials, should be recognized in the Acknowledgements section rather than in the Financial Disclosure.

b) State the initials, alongside each funding source, of each author to receive each grant. For example: “This work was supported by the National Institutes of Health (####### to AM; ###### to CJ) and the National Science Foundation (###### to AM).”
We have added initials to the initial statement. The funding agency requires the language and format in which we have used.

c) State what role the funders took in the study. If the funders had no role in your study, please state: “The funders had no role in study design, data collection and analysis, decision to publish, or preparation of the manuscript.”
We have now clarified and stated the funders role in the study (none; indicated the quoted statement).

For more information, please go to our submission guidelines:
[https://journals.plos.org/mentalhealth/s/submission-guidelines#loc-financial-disclosure-statement](https://nam12.safelinks.protection.outlook.com/?url=https%3A%2F%2Furldefense.com%2Fv3%2F__https%3A%2Fjournals.plos.org%2Fmentalhealth%2Fs%2Fsubmission-guidelines*loc-financial-disclosure-statement__%3BIw!!JqxBPMk!iAhEzj6Jj3TOFr8zDPgb9n0Qq8MTzU9Z3SaiZbtU9XlinDqUF6eV0GRgM1rspzhvtO5wsQNeDIiKIElOzys%24&data=05%7C02%7Cdwaltzma%40iu.edu%7C8aaf4d06503241e9958e08dece100411%7C1113be34aed14d00ab4bcdd02510be91%7C1%7C0%7C639174765432045009%7CUnknown%7CTWFpbGZsb3d8eyJFbXB0eU1hcGkiOnRydWUsIlYiOiIwLjAuMDAwMCIsIlAiOiJXaW4zMiIsIkFOIjoiTWFpbCIsIldUIjoyfQ%3D%3D%7C0%7C%7C%7C&sdata=OJFxkRO%2BtCFoehuvpaxs0nu%2FTz0UtFoZR9Kyar9seZA%3D&reserved=0)

2. Please ensure that the funders and grant numbers match between the Financial Disclosure field and the Funding Information tab in your submission form. Note that the funders must be provided in the same order in both places as well.

We have now ensured all funding information aligns with the financial disclosure form and in the same order.

3. Please send a completed 'Competing Interests' statement, including any COIs declared by your co-authors. If you have no competing interests to declare, please state “The authors have declared that no competing interests exist". Otherwise please declare all competing interests beginning with the statement "I have read the journal's policy and the authors of this manuscript have the following competing interests:
We have revised the Competing Interests Statement to align with journal guidelines.

For more information, please go to our submission guidelines:
[https://journals.plos.org/mentalhealth/s/submission-guidelines#loc-competing-interests](https://nam12.safelinks.protection.outlook.com/?url=https%3A%2F%2Furldefense.com%2Fv3%2F__https%3A%2Fjournals.plos.org%2Fmentalhealth%2Fs%2Fsubmission-guidelines*loc-competing-interests__%3BIw!!JqxBPMk!iAhEzj6Jj3TOFr8zDPgb9n0Qq8MTzU9Z3SaiZbtU9XlinDqUF6eV0GRgM1rspzhvtO5wsQNeDIiK2yZrss0%24&data=05%7C02%7Cdwaltzma%40iu.edu%7C8aaf4d06503241e9958e08dece100411%7C1113be34aed14d00ab4bcdd02510be91%7C1%7C0%7C639174765432067323%7CUnknown%7CTWFpbGZsb3d8eyJFbXB0eU1hcGkiOnRydWUsIlYiOiIwLjAuMDAwMCIsIlAiOiJXaW4zMiIsIkFOIjoiTWFpbCIsIldUIjoyfQ%3D%3D%7C0%7C%7C%7C&sdata=3ev1dhYudZT000eGs%2Bdg2IGFoy0wSLn0f1CeqWFN0pM%3D&reserved=0)

4. In the online submission form, you indicated that your data is available only on request from a third party. Please note that your Data Availability Statement is currently missing contact details for the third party, such as an email address or a link to where data requests can be made. Please update your statement with the missing information.
We have now added the weblink to the data repository to access these data.

5. Please provide separate main figure files in .tif or .eps format only and remove any figures embedded in your manuscript file. Please also ensure that all files are under our size limit of 10MB. Please leave the figure captions in the manuscript.
All figures have been removed from the manuscript and separately uploaded now.

For more information about how to convert your figure files please see our guidelines: [https://journals.plos.org/mentalhealth/s/figures](https://nam12.safelinks.protection.outlook.com/?url=https%3A%2F%2Furldefense.com%2Fv3%2F__https%3A%2Fjournals.plos.org%2Fmentalhealth%2Fs%2Ffigures__%3B!!JqxBPMk!iAhEzj6Jj3TOFr8zDPgb9n0Qq8MTzU9Z3SaiZbtU9XlinDqUF6eV0GRgM1rspzhvtO5wsQNeDIiKp1T_Uks%24&data=05%7C02%7Cdwaltzma%40iu.edu%7C8aaf4d06503241e9958e08dece100411%7C1113be34aed14d00ab4bcdd02510be91%7C1%7C0%7C639174765432090388%7CUnknown%7CTWFpbGZsb3d8eyJFbXB0eU1hcGkiOnRydWUsIlYiOiIwLjAuMDAwMCIsIlAiOiJXaW4zMiIsIkFOIjoiTWFpbCIsIldUIjoyfQ%3D%3D%7C0%7C%7C%7C&sdata=hk%2Byxb10sQkpfn%2FD5nYBXSO%2BrOAHiS4DiClcLjN%2BVpQ%3D&reserved=0)

6. We notice that your supplementary table is included in the manuscript file. Please remove them and upload them with the file type 'Supporting Information'. Please ensure that each Supporting Information file has a legend listed in the manuscript before or after the references list.
The supplementary table is now removed from the manuscript and uploaded as a separate document, along with Supplemental table header and legend after the references.

7. If the reviewer comments include a recommendation to cite specific previously published works, please review and evaluate these publications to determine whether they are relevant and should be cited. There is no requirement to cite these works unless the editor has indicated otherwise.
Thank you for mentioning; this situation did not occur.

Reviewers' comments:

Reviewer's Responses to Questions

**Comments to the Author**

1. Does this manuscript meet PLOS Mental Health’s [publication criteria](https://nam12.safelinks.protection.outlook.com/?url=https%3A%2F%2Furldefense.com%2Fv3%2F__https%3A%2Ftrack.editorialmanager.com%2FCL0%2Fhttps%3A*2F*2Fjournals.plos.org*2Fmentalhealth*2Fs*2Fcriteria-for-publication%2F1%2F010f019eddcc4d80-2a9dd4f6-7032-4237-b81c-a82411146912-000000%2FQv8qe5ABCkUUsIAjD2q5Y_iHtOaCL5sU4A_fHYg-Ag0%3D258__%3BJSUlJSU!!JqxBPMk!iAhEzj6Jj3TOFr8zDPgb9n0Qq8MTzU9Z3SaiZbtU9XlinDqUF6eV0GRgM1rspzhvtO5wsQNeDIiK9oQQL64%24&data=05%7C02%7Cdwaltzma%40iu.edu%7C8aaf4d06503241e9958e08dece100411%7C1113be34aed14d00ab4bcdd02510be91%7C1%7C0%7C639174765432116811%7CUnknown%7CTWFpbGZsb3d8eyJFbXB0eU1hcGkiOnRydWUsIlYiOiIwLjAuMDAwMCIsIlAiOiJXaW4zMiIsIkFOIjoiTWFpbCIsIldUIjoyfQ%3D%3D%7C0%7C%7C%7C&sdata=%2FkelgxpMp0etWmWBNLnZZQUnhSlmsaqaHBjLvZAGxnU%3D&reserved=0)? Is the manuscript technically sound, and do the data support the conclusions? The manuscript must describe methodologically and ethically rigorous research with conclusions that are appropriately drawn based on the data presented.

Reviewer #1: Yes

Reviewer #2: Yes

2. Has the statistical analysis been performed appropriately and rigorously?

Reviewer #1: Yes

Reviewer #2: Yes

3. Have the authors made all data underlying the findings in their manuscript fully available (please refer to the Data Availability Statement at the start of the manuscript PDF file)?

The [PLOS Data policy](https://nam12.safelinks.protection.outlook.com/?url=https%3A%2F%2Furldefense.com%2Fv3%2F__https%3A%2Ftrack.editorialmanager.com%2FCL0%2Fhttps%3A*2F*2Fjournals.plos.org*2Fmentalhealth*2Fs*2Fdata-availability%2F1%2F010f019eddcc4d80-2a9dd4f6-7032-4237-b81c-a82411146912-000000%2FEbnkyyXQGBQFtpqtgMF-b9c-JKF7nThLd85t81ryAM4%3D258__%3BJSUlJSU!!JqxBPMk!iAhEzj6Jj3TOFr8zDPgb9n0Qq8MTzU9Z3SaiZbtU9XlinDqUF6eV0GRgM1rspzhvtO5wsQNeDIiKmC6HPqs%24&data=05%7C02%7Cdwaltzma%40iu.edu%7C8aaf4d06503241e9958e08dece100411%7C1113be34aed14d00ab4bcdd02510be91%7C1%7C0%7C639174765432142479%7CUnknown%7CTWFpbGZsb3d8eyJFbXB0eU1hcGkiOnRydWUsIlYiOiIwLjAuMDAwMCIsIlAiOiJXaW4zMiIsIkFOIjoiTWFpbCIsIldUIjoyfQ%3D%3D%7C0%7C%7C%7C&sdata=o4uB59Zrqt8CWLgYQEo%2Bwd66Jm8o%2FD5LXEo%2B8T1uI84%3D&reserved=0) requires authors to make all data underlying the findings described in their manuscript fully available without restriction, with rare exception. The data should be provided as part of the manuscript or its supporting information, or deposited to a public repository. For example, in addition to summary statistics, the data points behind means, medians and variance measures should be available. If there are restrictions on publicly sharing data—e.g. participant privacy or use of data from a third party—those must be specified.

Reviewer #1: No
Thank you for bringing this to our attention. We have now provided a link.

Reviewer #2: Yes

4. Is the manuscript presented in an intelligible fashion and written in standard English?

PLOS Mental Health does not copyedit accepted manuscripts, so the language in submitted articles must be clear, correct, and unambiguous. Any typographical or grammatical errors should be corrected at revision, so please note any specific errors here.

Reviewer #1: Yes

Reviewer #2: Yes

5. Review Comments to the Author

**Reviewer #1:**

This manuscript addresses an important and underexplored question: whether childhood mild traumatic brain injury (mTBI) is associated with later-life mental and behavioral health outcomes among Veterans. Using data from the LIMBIC-CENC cohort, the authors leverage a large, well-characterized sample with rigorous mTBI adjudication and a broad set of outcome measures. The topic has clear clinical and public health relevance, particularly for understanding potential pre-enlistment factors shaping long-term outcomes in service members and Veterans. With appropriate refinement, this work has the potential to make a meaningful contribution to the literature and inform future research aimed at better supporting these populations.
Thank you for this comment.

The statistical approach is generally appropriate, and the dataset is a major strength. However, the manuscript exhibits a recurring pattern of inferential overextension and conceptual compression, where:
• associations are interpreted as implying causal structure
• heterogeneous constructs are treated as a unified domain
• sample-derived quantities are framed in population terms
• acknowledged limitations are not fully integrated into interpretation
These issues do not invalidate the analysis, but they materially affect interpretability, generalizability, and evidential strength. I recommend major revision.
We appreciate the points made by the reviewer. We will appropriately address these in the comments below.

1.General point.
Observational, retrospective exposure with cross-sectional outcomes is interpreted using causal language (“risk factor,” “lingering effects,” intervention implications), however, the analysis cannot distinguish causal effects from shared vulnerability, selection, or reporting processes. Given recall bias, residual confounding, and cohort selection, causal framing overstates evidential strength.
There are several issues to address here:
• Replace causal with associational language
• Explicitly acknowledge alternative explanations
• Reframe conclusions as identifying markers of increased risk
• Limit clinical implications to hypothesis generation
We thank the reviewer for this thoughtful and important observations. In response, we carefully reviewed the manuscript and revised language throughout, as well as ensured full acknowledgement of this in the limitations. We believe these revisions provide a more balanced interpretation of the findings while preserving the clinical relevance of the observed associations.

2. The study sample comes from a selected research cohort with specific inclusion criteria (e.g., combat exposure) and therefore does not represent all Veterans. As a result, these figures describe only the composition of this study sample, not how common childhood mTBI is in the wider Veteran population. Going forward, please reframe as cohort-specific proportions and clarify representativeness limits
We agree with the reviewer about this and have added text to the manuscript further describing our sample. We have made numerous statements throughout the methods, limitations, and overall to ensure reference is to the specific cohort examined and minimize broad Veteran population-based interpretations or implications.

3. An over lapping point, but distinct is the need to acknowledge whether the observed relationships themselves may be influenced by the sample selection who entered and remained in this cohort.
We agree with the reviewer about this and have added text to the manuscript.

4. Key early-life confounders (SES, adversity, behavioural traits) are not included, yet these plausibly affect both exposure and outcomes, leaving substantial residual confounding. Please explicitly acknowledge this, and clarify limits of adjustment
We agree with the reviewer about this and have added text.

5. The manuscript focuses on childhood mTBI while also adjusting for cumulative mTBI exposure, making it unclear whether observed associations reflect timing of injury or overall burden. This conflation complicates interpretation; the authors should clarify their conceptual model and discuss whether childhood mTBI provides information beyond cumulative exposure.
We thank the reviewer for bring up this point. However, we want to clarify that our primary analysis (Figure 1, S1 Table) does not adjust for cumulative mTBI burden. The data in this figure shows differences in outcomes between Veterans with mTBI in childhood and Veterans without mTBI in childhood. Therefore, these results do provide information independent of cumulative exposure. However, the data presented in Tables 2 and 3 are only within the mTBI in childhood group and do contain other variables of interest, including cumulative exposure to further examine associations within the cohort with mTBI. We believe the text in the Results and Discussion are appropriate to the data shown.

6. The manuscript positions itself as examining childhood mTBI but does not clearly situate its findings relative to prior analyses, particularly those (including likely earlier work in the LIMBIC-CENC cohort or related literature) that focus on: military-service mTBI or lifetime/cumulative mTBI exposure. Without explicit comparison, it is unclear whether childhood mTBI provides independent or incremental information, or the findings largely reflect known associations with cumulative mTBI burden. This creates ambiguity in the manuscript’s novelty and conceptual contribution and reinforces the unresolved tension between timing of exposure (childhood) vs dose/accumulation (multiple mTBIs). Please address in Introduction and or Discussion.
We have expanded the introduction and discussion to better address the current gaps in the literature and better describe how these findings may help address this gap.

7. No clear reporting of initial cohort size, exclusions, or missing data handling, which limits evaluation of bias and reproducibility. Please provide flow diagram, and report missingness and handling
We thank the reviewer for raising these points and have added them to the Methods and Results section of the manuscript. Missingness has been added the results section tables, and in the Methods section we clarified that missing data were handled using complete-case (case-wise deletion) analysis. The sample size for each multivariable model is provided as well.

8. Adult variables (education, BMI, physical activity) are used for adjustment, but this may introduce post-treatment bias and overadjustment. Please clarify causal roles and acknowledge potential bias.
We have acknowledged this in the limitations section. Due to the purpose of our study (i.e., exploratory associative analysis) and edits made through this revision, we believe it is much clearer to readers that these findings cannot and should not be used as causal effects.

9. The manuscript evaluates a range of outcomes that differ in measurement scale, recall period, and underlying construct (e.g., symptoms, behaviours, and traits), yet interprets them collectively as a unified “mental and behavioural health” domain. This heterogeneity limits comparability and risks overgeneralization. Please can the authors acknowledge these differences and interpret findings within specific domains rather than as a single coherent construct.
We agree with the reviewer and added text that is more explicit to the manuscript.

10. Numerous outcomes tested without correction or discussion. Please either apply correction or frame findings as exploratory. It may also be worth looking at pattern consistency across outcomes.
We agree with the reviewer and have framed this study as exploratory.

11. Limitations listed but not carried into interpretive claims. Please integrate limitations into Discussion and constrain conclusions on uncertainty
We respectfully disagree with this point. We think it is easier for a reader to understand the limitations of our study in a specific paragraph and thus wish to keep this text as is. The nature of our discussion and conclusions do not use causal language or suggest our effect estimates should be interpreted as such, and therefore this is inherently engrained in the discussion and further reminded to readers via the dedicated limitations paragraph. We have, however, constrained conclusions to uncertainty in the manuscript.

12. The manuscript extends its findings to suggest screening and intervention strategies, which implies a level of actionable evidence not supported by the observational, cross-sectional design. These implications should be tempered and reframed as hypothesis-generating, highlighting the need for further research rather than direct clinical application.
We thank the reviewer for raising these points and have edited the manuscript accordingly.

13. Physical activity and education framed as protective, but this may reflect reverse causation or underlying advantage. Please acknowledge alternative explanations.
Thank you for this important observation. We agree that the cross-sectional and observational nature of our study precludes causal inference regarding the associations between educational attainment, physical activity, and mental and behavioral health outcomes. We have revised the Discussion to acknowledge alternative explanations, including reverse causation and the potential influence of unmeasured factors such as premorbid cognitive ability, socioeconomic advantage, and access to health-promoting resources. We also added language emphasizing the need for longitudinal studies to clarify the directionality and causal mechanisms underlying these associations.

14. The Discussion primarily interprets findings along a causal pathway from childhood mTBI to later outcomes without adequately considering alternative explanations which risks biasing interpretation. Please can the authors explicitly acknowledge competing models, such as shared underlying vulnerability, early-life adversity, and potential reporting biases.
We thank the reviewer for this important observation. We agree that the cross-sectional and observational nature of our study does not permit causal inference and have revised the Discussion accordingly while adding in alternative explanations.

15. Limited discussion of how sample differs from broader populations. Expand generalizability discussion and wider implications of external validity.
Thank you for this suggestion. We expanded the limitations section to more fully discuss external validity.

16. Substance use findings differ but are not discussed, which weakens interpretive coherence.
Thank you for this observation. We agree that the absence of significant differences in alcohol and drug use outcomes warranted further discussion. We have added a paragraph to the Discussion.

17. The manuscript focuses primarily on statistical significance when interpreting results, with limited consideration of effect size magnitude. Some associations, while statistically robust, may be modest in practical terms; providing clinical context or benchmarks would help clarify their real-world relevance.

We originally chose, and respectively decline, to interpret these findings relevant to clinical thresholds or effect size magnitudes (i.e., unstandardized or standardized effect sizes) due to 1) numerous assessments examined have effect size magnitudes that vary in what is considered meaningful in the literature, 2) some do not have established thresholds for clinical meaningfulness which poses a quasi-objective challenge for us to interpret, and 3) interpreting these with clinical context or linkage begins approaching on the concerns of causal claims or the noted concern of “Limit clinical implications to hypothesis generation”.

18. Longitudinal cohort used, but analysis is cross-sectional. This may imply temporal inference. Please explicitly clarify.
Thank you for raising this point. We have clarified the cross-sectional nature of the study throughout the manuscript.

Moderate / Minor Issues
1. Limited Detail on Behavioral Risk Factor Surveillance System Variables: Clarify derivation and thresholds.
The BRFSS itself has been referenced and the variables for smoking and chewing dip use have been described in how they were categorized in line with CDC use.

2. Pittsburgh Sleep Quality Index Scoring: Clarify component-based scoring.
We have now clarified the 7 component scores captured in the PSQI.

3. Role of Kruskal–Wallis test: Clarify descriptive role.
Thank you for pointing this out. We didn’t use the Kruskal-Wallis test here. We have clarified the rationale for selecting each statistical test in the Methods:

Continuous variables with approximately symmetric distributions were compared using the Welch two-sample *t*-test. Variables with markedly skewed distributions were compared using the Wilcoxon rank-sum test. Categorical variables were compared using the Pearson chi-square test or Fisher's exact test when any expected cell count was <5.

4. Model Choice Explanation: Clarify rationale and interpretation.
In the Methods section we attempted to provide more details on how each model was selected and how to interpret results:

In this cross-sectional analysis, separate multivariable linear, gamma, quasi-Poisson, or ordinal logistic regression models were used to estimate the association between lifetime childhood mTBI group and each mental and behavioral health outcome, with model selection based on the distributional characteristics of the outcome. Most outcomes (anxiety, emotional behavioral dyscontrol score, neurobehavioral symptoms score, satisfaction with life score, and sleep quality score) were analyzed using linear regression, with results reported as mean differences and 95% confidence intervals (CI). For non-normally distributed outcomes, the modified Park test was used to help identify an appropriate variance function and guide selection among candidate generalized linear models.(49). Gamma regression with a log link was used for anger and resilience, which were positively skewed with variances increasing with the mean, and results are reported as mean ratios (MR). Quasi-Poisson regression with a log link was used for PTSD symptoms, depression severity, and alcohol use score because these outcomes exhibited overdispersion (variance exceeding the mean); results are also reported as MR. Drug use severity was analyzed using ordinal logistic regression, with results reported as odds ratios (OR). The proportional odds assumption was assessed for the multivariable model for drug use severity and was found to be satisfied.

5. Ordinal Logistic Assumptions: State whether tested.
We have modified the methods section to include: The proportional odds assumption was assessed for the multivariable model for drug use severity and was found to be satisfied.

6. Coarse Exposure Definition: Acknowledge heterogeneity.
It is unclear what specific exposure metric is explicitly meant here. We are presuming the mTBI variable and specifically childhood mTBI. If so, we have thoroughly described it in the methods, reported the descriptive which highlight the heterogeneity, and lastly described in the discussion and limitations interpretation considerations for this variable.

7. Reproducibility and transparency: Provide web address for data access and include statement that R scripts are available at the same location, where permissible.
All reproducibility and transparency information has been updated in line with PLOS policies; we provide the specific weblink to initiate data access from the federal data repository.

Final Synthesis
The manuscript is methodologically competent and based on a strong dataset, but exhibits a consistent pattern:
Uncertainty is acknowledged but not allowed to meaningfully constrain inference.
Addressing this would align conclusions with the limits of the design, improve interpretability, and strengthen the manuscript’s contribution to research supporting service personnel and Veterans.

 We thank the reviewer for the thoughtful comments and have addressed each one point by point.

**Reviewer #2**

This manuscript addresses an important and clinically relevant topic by investigating the long-term association between childhood mild traumatic brain injury (mTBI) and mental and behavioral health outcomes in a large cohort of U.S. Veterans. The large sample size, use of a well-characterized cohort, and comprehensive assessment of multiple psychological outcomes represent important strengths.
However, some methodological and interpretative issues should be addressed before the manuscript is suitable for publication.
We thank the reviewer for their thoughtful evaluation of our manuscript and for recognizing the importance of the research question, the strengths of the study cohort, and the comprehensive assessment of mental and behavioral health outcomes. We appreciate the reviewer’s constructive feedback regarding the methodological and interpretative aspects of the manuscript. In response, we have carefully revised the manuscript to address all concerns raised. Detailed responses to each comment are provided below.

Main revisions
• Although the models adjust for several demographic and behavioral variables, important pre-injury confounders (e.g., childhood psychiatric disorders, adverse childhood experiences, socioeconomic status, family environment) are not considered. These factors may predispose individuals both to childhood mTBI and poorer adult mental health, limiting causal interpretation.

We agree with this point and have added this as a limitation.

• Excluding participants with adult-only mTBI substantially narrows the comparison and may introduce selection bias. The authors should discuss how this exclusion may affect generalizability and interpretation of the findings.

Thank you for raising this point. We have added this as a limitation.

• Although the LIMBIC-CENC is a prospective longitudinal study, the present analysis is cross-sectional. This distinction should be emphasized more clearly throughout the manuscript, avoiding language that could imply temporal or causal relationships.

Thank you for this suggestion. We have added this clarity throughout the manuscript.

• Numerous outcomes were analyzed without adjustment for multiple testing. The authors should justify this approach or provide a sensitivity analysis controlling for multiple comparisons.
We thank the reviewer for this important comment. The primary aim of this study was exploratory in nature and intended to identify potential associations between childhood mTBI and a broad range of mental and behavioral health outcomes among Veterans with combat exposure. Because the outcomes examined represent distinct clinical domains rather than multiple tests of a single hypothesis, we elected not to apply a formal multiple-comparison correction in the primary analyses. We acknowledge that this approach increases the risk of Type I error and have added text to the manuscript clarifying the exploratory nature of the study and emphasizing that the findings should be interpreted cautiously and considered hypothesis-generating. We have also expanded the Limitations section to note that the large number of statistical comparisons may increase the likelihood of chance findings and that replication in independent cohorts is needed to confirm the observed associations.

Minor comments
• The Introduction could better distinguish the effects of childhood mTBI from those of military-related mTBI, thereby strengthening the rationale for focusing specifically on pre-military injuries.

We thank the reviewer for this helpful suggestion and have added text to the Introduction.

• More details regarding the severity, mechanism, and frequency of childhood mTBIs would improve the characterization of the exposure.

We have added mechanism and frequency of childhood mTBIs to Table 1. The severity of injury are all mild.
