# Supplementary material for: Association of childhood mild traumatic brain injury with mental and behavioral health outcomes later in life among U.S. Veterans: A LIMBIC-CENC study
Source: PLOS Ment Health. 2026 Oct 21;3(10):e0000679. doi: 10.1371/journal.pmen.0000679 (PMC13686504; doi:10.1371/journal.pmen.0000679)
Supplement: Peer review history

# Author comment: Author response to Decision Letter 0

**DOI:** 10.1371/journal.pmen.0000679.r001

**Transfer Alert**

This paper was transferred from another journal. As a result, its full editorial history (including decision letters, peer reviews and author responses) may not be present.

20 Apr 2026

# Peer review: Decision Letter 0

**DOI:** 10.1371/journal.pmen.0000679.r002

**Authors:** Elisa Di Rosa

18 Jun 2026

PMEN-D-26-00222

Association of Childhood Mild Traumatic Brain Injury with Mental and Behavioral Health Outcomes Later in Life Among U.S. Veterans: A LIMBIC-CENC Study

PLOS Mental Health

Dear Dr. Landon

Thank you for submitting your manuscript to PLOS Mental Health. After careful consideration, we feel that it has merit but does not fully meet PLOS Mental Health’s publication criteria as it currently stands. Therefore, we invite you to submit a revised version of the manuscript that addresses the points raised during the review process.

Please submit your revised manuscript by July 18th. If you will need more time than this to complete your revisions, please reply to this message or contact the journal office at mentalhealth@plos.org. When you're ready to submit your revision, log on to https://www.editorialmanager.com/pmen/ and select the 'Submissions Needing Revision' folder to locate your manuscript file.

Please include the following items when submitting your revised manuscript:

- A letter that responds to each point raised by the editor and reviewer(s). You should upload this letter as a separate file labeled 'Response to Reviewers'.
- A marked-up copy of your manuscript that highlights changes made to the original version. You should upload this as a separate file labeled 'Revised Manuscript with Track Changes'.
- An unmarked version of your revised paper without tracked changes. You should upload this as a separate file labeled 'Manuscript'.

Guidelines for resubmitting your figure files are available below the reviewer comments at the end of this letter.

As the corresponding author, your ORCID iD is verified in the submission system and will appear in the published article. PLOS supports the use of ORCID, and we encourage all coauthors to register for an ORCID iD and use it as well. Please encourage your coauthors to verify their ORCID iD within the submission system before final acceptance, as unverified ORCID iDs will not appear in the published article. *Only*  the individual author can complete the verification step; PLOS staff *cannot*  verify ORCID iDs on behalf of authors.

We look forward to receiving your revised manuscript.

Kind regards,

Elisa Di Rosa

Academic Editor

PLOS Mental Health

Journal Requirements:

1. Please provide a detailed online Financial Disclosure statement. This is published with the article. It must therefore be completed in full sentences and contain the exact wording you wish to be published.

a) Please clarify all sources of financial support for your study. List the grants, grant numbers, and organizations that funded your study, including funding received from your institution. Please note that suppliers of material support, including research materials, should be recognized in the Acknowledgements section rather than in the Financial Disclosure.

b) State the initials, alongside each funding source, of each author to receive each grant. For example: “This work was supported by the National Institutes of Health (####### to AM; ###### to CJ) and the National Science Foundation (###### to AM).”

c) State what role the funders took in the study. If the funders had no role in your study, please state: “The funders had no role in study design, data collection and analysis, decision to publish, or preparation of the manuscript.”

For more information, please go to our submission guidelines:

https://journals.plos.org/mentalhealth/s/submission-guidelines#loc-financial-disclosure-statement

2. Please ensure that the funders and grant numbers match between the Financial Disclosure field and the Funding Information tab in your submission form. Note that the funders must be provided in the same order in both places as well.

3. Please send a completed 'Competing Interests' statement, including any COIs declared by your co-authors. If you have no competing interests to declare, please state “The authors have declared that no competing interests exist". Otherwise please declare all competing interests beginning with the statement "I have read the journal's policy and the authors of this manuscript have the following competing interests:

For more information, please go to our submission guidelines:

https://journals.plos.org/mentalhealth/s/submission-guidelines#loc-competing-interests

4. In the online submission form, you indicated that your data is available only on request from a third party. Please note that your Data Availability Statement is currently missing contact details for the third party, such as an email address or a link to where data requests can be made. Please update your statement with the missing information.

5. Please provide separate main figure files in .tif or .eps format only and remove any figures embedded in your manuscript file. Please also ensure that all files are under our size limit of 10MB. Please leave the figure captions in the manuscript.

For more information about how to convert your figure files please see our guidelines: https://journals.plos.org/mentalhealth/s/figures

6. We notice that your supplementary table is included in the manuscript file. Please remove them and upload them with the file type 'Supporting Information'. Please ensure that each Supporting Information file has a legend listed in the manuscript before or after the references list.

7. If the reviewer comments include a recommendation to cite specific previously published works, please review and evaluate these publications to determine whether they are relevant and should be cited. There is no requirement to cite these works unless the editor has indicated otherwise.

Reviewers' comments:

Reviewer's Responses to Questions

**Comments to the Author**

1. Does this manuscript meet PLOS Mental Health’s publication criteria?

Reviewer #1: Yes

Reviewer #2: Yes

**********

2. Has the statistical analysis been performed appropriately and rigorously?

Reviewer #1: Yes

Reviewer #2: Yes

**********

3. Have the authors made all data underlying the findings in their manuscript fully available (please refer to the Data Availability Statement at the start of the manuscript PDF file)?

The PLOS Data policy

Reviewer #1: No

Reviewer #2: Yes

**********

4. Is the manuscript presented in an intelligible fashion and written in standard English?

Reviewer #1: Yes

Reviewer #2: Yes

**********

**Reviewer #1:**

This manuscript addresses an important and underexplored question: whether childhood mild traumatic brain injury (mTBI) is associated with later-life mental and behavioral health outcomes among Veterans. Using data from the LIMBIC-CENC cohort, the authors leverage a large, well-characterized sample with rigorous mTBI adjudication and a broad set of outcome measures.

The topic has clear clinical and public health relevance, particularly for understanding potential pre-enlistment factors shaping long-term outcomes in service members and Veterans. With appropriate refinement, this work has the potential to make a meaningful contribution to the literature and inform future research aimed at better supporting these populations.

The statistical approach is generally appropriate, and the dataset is a major strength. However, the manuscript exhibits a recurring pattern of inferential overextension and conceptual compression, where:

• associations are interpreted as implying causal structure

• heterogeneous constructs are treated as a unified domain

• sample-derived quantities are framed in population terms

• acknowledged limitations are not fully integrated into interpretation

These issues do not invalidate the analysis, but they materially affect interpretability, generalizability, and evidential strength. I recommend major revision.

1.General point.

Observational, retrospective exposure with cross-sectional outcomes is interpreted using causal language (“risk factor,” “lingering effects,” intervention implications), however, the analysis cannot distinguish causal effects from shared vulnerability, selection, or reporting processes. Given recall bias, residual confounding, and cohort selection, causal framing overstates evidential strength.

There are several issues to address here:

• Replace causal with associational language

• Explicitly acknowledge alternative explanations

• Reframe conclusions as identifying markers of increased risk

• Limit clinical implications to hypothesis generation

2. The study sample comes from a selected research cohort with specific inclusion criteria (e.g., combat exposure) and therefore does not represent all Veterans. As a result, these figures describe only the composition of this study sample, not how common childhood mTBI is in the wider Veteran population. Going forward, please reframe as cohort-specific proportions and clarify representativeness limits

3. An over lapping point, but distinct is the need to acknowledge whether the observed relationships themselves may be influenced by the sample selection who entered and remained in this cohort.

4. Key early-life confounders (SES, adversity, behavioural traits) are not included, yet these plausibly affect both exposure and outcomes, leaving substantial residual confounding. Please explicitly acknowledge this, and clarify limits of adjustment

5. The manuscript focuses on childhood mTBI while also adjusting for cumulative mTBI exposure, making it unclear whether observed associations reflect timing of injury or overall burden. This conflation complicates interpretation; the authors should clarify their conceptual model and discuss whether childhood mTBI provides information beyond cumulative exposure.

6. The manuscript positions itself as examining childhood mTBI but does not clearly situate its findings relative to prior analyses, particularly those (including likely earlier work in the LIMBIC-CENC cohort or related literature) that focus on: military-service mTBI or lifetime/cumulative mTBI exposure. Without explicit comparison, it is unclear whether childhood mTBI provides independent or incremental information, or the findings largely reflect known associations with cumulative mTBI burden. This creates ambiguity in the manuscript’s novelty and conceptual contribution and reinforces the unresolved tension between timing of exposure (childhood) vs dose/accumulation (multiple mTBIs). Please address in Introduction and or Discussion.

7. No clear reporting of initial cohort size, exclusions, or missing data handling, which limits evaluation of bias and reproducibility. Please provide flow diagram, and report missingness and handling

8. Adult variables (education, BMI, physical activity) are used for adjustment, but this may introduce post-treatment bias and overadjustment. Please clarify causal roles and acknowledge potential bias.

9. The manuscript evaluates a range of outcomes that differ in measurement scale, recall period, and underlying construct (e.g., symptoms, behaviours, and traits), yet interprets them collectively as a unified “mental and behavioural health” domain. This heterogeneity limits comparability and risks overgeneralization. Please can the authors acknowledge these differences and interpret findings within specific domains rather than as a single coherent construct.

10. Numerous outcomes tested without correction or discussion. Please either apply correction or frame findings as exploratory. It may also be worth looking at pattern consistency across outcomes.

11. Limitations listed but not carried into interpretive claims. Please integrate limitations into Discussion and constrain conclusions on uncertainty

12. The manuscript extends its findings to suggest screening and intervention strategies, which implies a level of actionable evidence not supported by the observational, cross-sectional design. These implications should be tempered and reframed as hypothesis-generating, highlighting the need for further research rather than direct clinical application.

13. Physical activity and education framed as protective, but this may reflect reverse causation or underlying advantage. Please acknowledge alternative explanations.

14. The Discussion primarily interprets findings along a causal pathway from childhood mTBI to later outcomes without adequately considering alternative explanations which risks biasing interpretation. Please can the authors explicitly acknowledge competing models, such as shared underlying vulnerability, early-life adversity, and potential reporting biases.

15. Limited discussion of how sample differs from broader populations. Expand generalizability discussion and wider implications of external validity.

16. Substance use findings differ but are not discussed, which weakens interpretive coherence.

17. The manuscript focuses primarily on statistical significance when interpreting results, with limited consideration of effect size magnitude. Some associations, while statistically robust, may be modest in practical terms; providing clinical context or benchmarks would help clarify their real-world relevance.

18. Longitudinal cohort used, but analysis is cross-sectional. This may imply temporal inference. Please explicitly clarify.

Moderate / Minor Issues

1. Limited Detail on Behavioral Risk Factor Surveillance System Variables: Clarify derivation and thresholds.

2. Pittsburgh Sleep Quality Index Scoring: Clarify component-based scoring.

3. Role of Kruskal–Wallis test: Clarify descriptive role.

4. Model Choice Explanation: Clarify rationale and interpretation.

5. Ordinal Logistic Assumptions: State whether tested.

6. Coarse Exposure Definition: Acknowledge heterogeneity.

7. Reproducibility and transparency: Provide web address for data access and include statement that R scripts are available at the same location, where permissible

Final Synthesis

The manuscript is methodologically competent and based on a strong dataset, but exhibits a consistent pattern:

Uncertainty is acknowledged but not allowed to meaningfully constrain inference.

Addressing this would align conclusions with the limits of the design, improve interpretability, and strengthen the manuscript’s contribution to research supporting service personnel and Veterans.

**Reviewer #2**

This manuscript addresses an important and clinically relevant topic by investigating the long-term association between childhood mild traumatic brain injury (mTBI) and mental and behavioral health outcomes in a large cohort of U.S. Veterans. The large sample size, use of a well-characterized cohort, and comprehensive assessment of multiple psychological outcomes represent important strengths.

However, some methodological and interpretative issues should be addressed before the manuscript is suitable for publication.

Main revisions

• Although the models adjust for several demographic and behavioral variables, important pre-injury confounders (e.g., childhood psychiatric disorders, adverse childhood experiences, socioeconomic status, family environment) are not considered. These factors may predispose individuals both to childhood mTBI and poorer adult mental health, limiting causal interpretation.

• Excluding participants with adult-only mTBI substantially narrows the comparison and may introduce selection bias. The authors should discuss how this exclusion may affect generalizability and interpretation of the findings.

• Although the LIMBIC-CENC is a prospective longitudinal study, the present analysis is cross-sectional. This distinction should be emphasized more clearly throughout the manuscript, avoiding language that could imply temporal or causal relationships.

• Numerous outcomes were analyzed without adjustment for multiple testing. The authors should justify this approach or provide a sensitivity analysis controlling for multiple comparisons.

Minor comments

• The Introduction could better distinguish the effects of childhood mTBI from those of military-related mTBI, thereby strengthening the rationale for focusing specifically on pre-military injuries.

• More details regarding the severity, mechanism, and frequency of childhood mTBIs would improve the characterization of the exposure.

**********

what does this mean?). If published, this will include your full peer review and any attached files.

**Do you want your identity to be public for this peer review?**  If you choose “no”, your identity will remain anonymous but your review may still be made public.

For information about this choice, including consent withdrawal, please see our Privacy Policy

Reviewer #1: **Yes:**  Prof/Dr Nicky Edelstyn

Reviewer #2: **Yes:**  Isabella Giulia Franzoi

**********

# Author comment: Author response to Decision Letter 1

**DOI:** 10.1371/journal.pmen.0000679.r003

23 Jul 2026

Attachment Submitted filename: Response to Reviewers- 2026-07-14.docx

# Peer review: Decision Letter 1

**DOI:** 10.1371/journal.pmen.0000679.r004

**Authors:** Elisa Di Rosa

9 Sep 2026

Association of Childhood Mild Traumatic Brain Injury with Mental and Behavioral Health Outcomes Later in Life Among U.S. Veterans: A LIMBIC-CENC Study

PMEN-D-26-00222R1

Dear Dr Landon B. Lempke

We are pleased to inform you that your manuscript 'Association of Childhood Mild Traumatic Brain Injury with Mental and Behavioral Health Outcomes Later in Life Among U.S. Veterans: A LIMBIC-CENC Study' has been provisionally accepted for publication in PLOS Mental Health.

Before your manuscript can be formally accepted you will need to complete some formatting changes, which you will receive in a follow up email. A member of our team will be in touch with a set of requests.

Please note that your manuscript will not be scheduled for publication until you have made the required changes, so a swift response is appreciated.

IMPORTANT: The editorial review process is now complete. PLOS will only permit corrections to spelling, formatting or significant scientific errors from this point onwards. Requests for major changes, or any which affect the scientific understanding of your work, will cause delays to the publication date of your manuscript.

If your institution or institutions have a press office, please notify them about your upcoming paper to help maximize its impact. If they'll be preparing press materials, please inform our press team as soon as possible -- no later than 48 hours after receiving the formal acceptance. Your manuscript will remain under strict press embargo until 2 pm Eastern Time on the date of publication. For more information, please contact mentalhealth@plos.org.

Thank you again for supporting Open Access publishing; we are looking forward to publishing your work in PLOS Mental Health.

Best regards,

Elisa Di Rosa

Academic Editor

PLOS Mental Health

***********************************************************

Reviewer Comments (if any, and for reference):

Reviewer's Responses to Questions

**Comments to the Author**

Reviewer #1: All comments have been addressed

Reviewer #2: All comments have been addressed

**********

publication criteria?

Reviewer #1: Yes

Reviewer #2: Yes

**********

3. Has the statistical analysis been performed appropriately and rigorously?

Reviewer #1: (No Response)

Reviewer #2: Yes

**********

4. Have the authors made all data underlying the findings in their manuscript fully available (please refer to the Data Availability Statement at the start of the manuscript PDF file)?

The PLOS Data policy

Reviewer #1: Yes

Reviewer #2: Yes

**********

5. Is the manuscript presented in an intelligible fashion and written in standard English?

Reviewer #1: Yes

Reviewer #2: Yes

**********

Reviewer #1: You have done a great job addressing my comments, and where push back is present, I am happy to go along with your suggestions.

Reviewer #2: Thank you for having addressed all my previous comments.

**********

what does this mean?). If published, this will include your full peer review and any attached files.

**Do you want your identity to be public for this peer review?**  If you choose “no”, your identity will remain anonymous but your review may still be made public.

For information about this choice, including consent withdrawal, please see our Privacy Policy

Reviewer #1: **Yes:**  Prof Nicky Edelstyn

Reviewer #2: **Yes:**  Isabella Giulia Franzoi

**********
